# From Animal Poisons and Venoms to Medicines: Achievements, Challenges and Perspectives in Drug Discovery

**DOI:** 10.3389/fphar.2020.01132

**Published:** 2020-07-24

**Authors:** Karla de Castro Figueiredo Bordon, Camila Takeno Cologna, Elisa Corrêa Fornari-Baldo, Ernesto Lopes Pinheiro-Júnior, Felipe Augusto Cerni, Fernanda Gobbi Amorim, Fernando Antonio Pino Anjolette, Francielle Almeida Cordeiro, Gisele Adriano Wiezel, Iara Aimê Cardoso, Isabela Gobbo Ferreira, Isadora Sousa de Oliveira, Johara Boldrini-França, Manuela Berto Pucca, Mateus Amaral Baldo, Eliane Candiani Arantes

**Affiliations:** ^1^ Laboratory of Animal Toxins, Department of BioMolecular Sciences, School of Pharmaceutical Sciences of Ribeirão Preto, University of São Paulo, Ribeirão Preto, Brazil; ^2^ Health and Science Institute, Paulista University, São José do Rio Pardo, Brazil; ^3^ Postgraduate Program in Pharmaceutical Sciences, Vila Velha University, Vila Velha, Brazil; ^4^ Department of Pharmacy, Federal Institute of Education, Science and Technology of Paraná, Palmas, Brazil; ^5^ Postgraduate Program in Ecosystem Ecology, Vila Velha University, Vila Velha, Brazil; ^6^ Medical School, Federal University of Roraima, Boa Vista, Brazil

**Keywords:** poison, venom, toxin, drug discovery, scorpion, snake, toad, *Conus*

## Abstract

Animal poisons and venoms are comprised of different classes of molecules displaying wide-ranging pharmacological activities. This review aims to provide an in-depth view of toxin-based compounds from terrestrial and marine organisms used as diagnostic tools, experimental molecules to validate postulated therapeutic targets, drug libraries, prototypes for the design of drugs, cosmeceuticals, and therapeutic agents. However, making these molecules applicable requires extensive preclinical trials, with some applications also demanding clinical trials, in order to validate their molecular target, mechanism of action, effective dose, potential adverse effects, as well as other fundamental parameters. Here we go through the pitfalls for a toxin-based potential therapeutic drug to become eligible for clinical trials and marketing. The manuscript also presents an overview of the current picture for several molecules from different animal venoms and poisons (such as those from amphibians, cone snails, hymenopterans, scorpions, sea anemones, snakes, spiders, tetraodontiformes, bats, and shrews) that have been used in clinical trials. Advances and perspectives on the therapeutic potential of molecules from other underexploited animals, such as caterpillars and ticks, are also reported. The challenges faced during the lengthy and costly preclinical and clinical studies and how to overcome these hindrances are also discussed for that drug candidates going to the bedside. It covers most of the drugs developed using toxins, the molecules that have failed and those that are currently in clinical trials. The article presents a detailed overview of toxins that have been used as therapeutic agents, including their discovery, formulation, dosage, indications, main adverse effects, and pregnancy and breastfeeding prescription warnings. Toxins in diagnosis, as well as cosmeceuticals and atypical therapies (bee venom and leech therapies) are also reported. The level of cumulative and detailed information provided in this review may help pharmacists, physicians, biotechnologists, pharmacologists, and scientists interested in toxinology, drug discovery, and development of toxin-based products.

## Introduction

Animal poisons and venoms are rich sources of proteins, peptides, neurotransmitters, among other compounds. Together, these molecules can induce major damages in the prey’s body, being one of the mechanisms employed by these animals to subdue and/or kill their preys or predators. The main difference between the terms “poison” and “venom” is the delivery method. Poisons are generated by specialized cells or tissues or are acquired from the diet, causing prey toxicity by ingestion or contact with the poisonous animal. On the other hand, venoms are produced by a tissue or organ (venom gland) and are parenterally introduced into the prey by the venomous animal, with a specialized apparatus (fang, stinger, teeth, nematocysts, among others) (Fox and Serrano, 2007). The word “toxin” will be used for both compounds from animal poisons and venoms in the whole article.

As a result of evolution and natural selection, toxins from animal poisons and venoms display wide-ranging pharmacological activities. Since the toxin targets are related to biological functions, with many of them playing important roles in human diseases, several venom components were used in the design of new therapeutic agents. They were also employed as cosmeceuticals, diagnostic tools, and experimental molecules to validate postulated therapeutic targets, improving several drug libraries (Ghosh et al., 2019; Utkin et al., 2019).

Although several biologically active toxins have been reported from terrestrial and marine organisms, there is a large gap between the initial drug discovery phase, including their validation as drug models, and their use in a clinical study. Drug candidates must pass through an extensive range of *in vitro* and *in vivo* tests to establish their pharmacology and biochemistry, carcinogenicity, and effects on the reproductive system, to assess their safety before moving on to the clinical phases (Tamimi and Ellis, 2009). In other words, drug development includes the discovery of a candidate molecule, preclinical and clinical studies, which are usually costly and takes a significant amount of time to attend the requirements stated by the regulatory agencies throughout the world.

This review aims to highlight the key successes and some examples of the obstacles and challenges faced when developing toxin-based drugs. It covers toxins from poisonous and venomous animals, drugs that target diverse pathological conditions, the molecules that have failed, and those that are currently in clinical trials. It also aims to encourage scientists to elucidate the mechanism of action of the already known venom components, discover new molecules with innovative therapeutic potential, and develop strategies to improve their pharmacokinetic and pharmacodynamic properties. Moreover, perspectives on the research and development of a wide range of toxins from several underexploited animal poisons and venoms are also discussed.

## Achievements With Animal Toxin-Based Molecules

Readers and scientists looking for approved drugs must consider the databases from regulatory agencies, such as the US Food and Drug Administration (FDA) and the European Medicines Agency (EMA). Furthermore, valuable information for health professionals and general public can be found at the Drug Information Database. However, the information provided by these databases is significantly limited, since biotechnology companies and pharmaceutical industries usually perform the drug development processes. Thus, much of the information relevant to drug development is not published and/or quite difficult to access.

Therefore, the subsections *Approved Drugs* to *Venom Therapies* will address the toxin-based approved drugs, diagnostic tools, cosmeceuticals and venom therapies, respectively, with the currently available details found at these databases.

### Approved Drugs

Among the 11 approved toxin-based molecules marketed, one molecule (ziconotide) is obtained from cone snails, two from lizards (exenatide and lixisenatide), two from leeches (bivalirudin and desirudin), and six from snakes (captopril, enalapril, tirofiban, eptifibatide, batroxobin, and cobratide). Batroxobin and cobratide are native compounds purified from snake venoms, desirudin is a recombinant molecule, and the other drugs (bivalirudin, captopril, enalapril, eptifibatide, exenatide, tirofiban, and ziconotide) are synthetic molecules (**Table 1**).

**Table 1:** Approved drugs and therapies for human use.

Molecule(brand name)	Species origin of venom toxin	Production	Formulation	Mechanism of action	Use	Dosage(maximum dose per day)*	More frequently reported adverse effects, pregnancy and breastfeeding warnings*	Reference
**Batroxobin (Defibrase^®^) ^(1)^**	Brazilian lancehead snake (*Bothrops moojeni*)	Purified from venom	Ampoule contains 10 batroxobin units, NaCl, chlorobutanol, and partially hydrolyzed gelatin in water	Cleaves Aα-chain of fibrinogen	Acute cerebral infarction; unspecific angina pectoris; sudden deafness	40 batroxobin units by i.v. infusion over 1 h.	Microvascular thrombosis	(Stocker, 1978; Vu et al., 2013; Pentapharm DSM Nutritional Products Ltd, 2018)
**Batroxobin (Plateltex-Act^®^) ^(1)^**	Common lancehead snake (*Bothrops atrox*)	Purified from venom	Vial of batroxobin (5 batroxobin units/1 ml), and 1 vial of calcium gluconate (940 mg gluconate/10 ml)	Cleaves Aα-chain of fibrinogen	Gelification of blood for topical applications	1 ml (or maximum of 1.5 ml) of calcium gluconate mixed with batroxobin (5 U). This mixture is mixed with 6-10 ml of platelet concentrate. After gel formation (7-10 min), it is applied on the area or in the site to be treated.	No toxicity phenomena are described in the tissues treated with the gel.	(Plateltex, 2018)
**Batroxobin - Fibrin sealant (Vivostat^®^) ^(1)^**	Brazilian lancehead snake (*Bothrops moojeni*)	Purified from venom	Medical device used for the preparation of an autologous fibrin; citrate	Cleaves Aα-chain of fibrinogen	Autologous fibrin sealant in surgery	Citrate is added to the device (during surgery or 24 h before), where is drawn 120 ml of the patient’s blood. After 25 min, an autologous fibrin is ready for use.	The sealant has no known adverse effects.	(Kjaergard and Trumbull, 1998; Vivostat A/S, 2018)
**Bee venom therapy (Apitox^®^)**	Honeybee *Apis mellifera*	Whole venom	100 µg/1 ml (bee venom in 0.9% NaCl)	Anti-inflammatory action; alteration of the immune response *via* antigen competition	Pain associated with osteoarthritis and multiple sclerosis	Monthly s.c. injections; twice weekly range from 1 to 20 intradermal injections (100 µg/0.1 ml saline)—at acupuncture points	Irritation, swollen, reddened skin and severe allergic reactions that can be life-threatening.	(Gotter, 2019; US National Library of Medicine, 2020)
**Bivalirudin (Angiomax^®^) ^(2)^**	European medicinal leech (*Hirudo medicinalis*)	Synthetic	Powder for injection, 250 mg, (bivalirudin trifluoroactetate, mannitol and sodium hidroxide)	Reversible direct thrombin inhibitor	Anticoagulant in percutaneous coronary intervention	0.75 mg/kg by direct IV injection, followed by 1.75 mg/kg per hour (300-325 mg daily)	Hemorragic events, back pain, pain (unspecified), nausea, headache, hyper/hypotension, injection site pain, insomnia, vomiting, pelvic pain, anxiety, bradycardia, dyspepsia, abdominal pain, fever, nervouness, urinary retention; pregnancy risk factor B	(US Food and Drug Administration, 2020)
**Captopril (Capoten^®^) ^(3)^**	Jararaca pit viper snake (*Bothrops jararaca*)	Synthetic	Oral tablets: 12.5, 25, 50, and 100 mg (inactive ingredients: anhydrous lactose, colloidal silicon dioxide, crospovidone, microcrystalline cellulose, and stearic acid)	Angiotensin-converting enzyme inhibitor	Hypertension, cardiac failure	50 or 100 mg orally 3 times a day (maximum dose: 450 mg/day)	Cough and skin rash; US FDA pregnancy category D; excreted into human milk—discontinue breastfeeding or discontinue the drug, since the effects in the nursing infant are unknown	(US Food and Drug Administration, 2020)
**Cobratide (Ketongning, cobrotoxin) ^(2)^**	Chinese cobra (*Naja naja atra*)	Purified from venom	Freeze-dried powder (70 or 140 μg/vial) with dextran and glycine as excipient, for injection and cobratide enteric coated capsule	Blockage of nicotinic receptors	Chronic arthralgia, sciatica, neuropathic headache	Minimum and maximum daily dose are 280 μg and 840 μg, respectively—enteric coated capsule (CN101381408B)	Fatal side effects, such as respiration inhibition, can occur when it is injected at higher dosage levels.	(Chen et al., 2016; Orientoxin Biotech Co. Ltd., 2019)
**Desirudin (Iprivask^®^) ^(2)^**	European medicinal leech (*Hirudo medicinalis*)	Recombinant	Sterile powder for injection (desirudin-15.75 mg, anhydrous magnesium chloride-1.31 mg, and sodium hydroxide for injection USP)	Selective and near-irreversible inhibitor of thrombin	Prevention of venous thrombotic events	15 mg (5-15 min prior surgery), followed by 15 mg every 12 h up to 12 days	Bleeding, deep vein thrombophlebitis, wound secretion, nausea, vomiting, fever, hematoma, anemia; pregnancy risk factor C; no breastfeeding when using desirudin	(US Food and Drug Administration, 2020)
**Enalapril (Vasotec^®^) ^(3)^**	Jararaca pit viper snake (*Bothrops jararaca*)	Synthetic	Oral Tablets, 2.5, 5, 10, and 20 mg; 1.25 mg/ml i.v. (with benzyl alcohol 0.9%)	Angiotensin-converting enzyme inhibitor	Hypertension, cardiac failure	2.5 mg twice daily up to 10-20 mg twice daily. Increased dosage up to 40 mg/day (1 or 2 divided doses)	Increased serum creatinine,hypotension, dizziness, headache, fatigue, skin rash, abdominal pain, anorexia, constipation, diarrhea, nausea, vomiting, cough, dyspnea; US FDA pregnancy category D; excreted into human milk—discontinue breastfeeding or discontinue the drug, since the effects in the nursing infant are unknown	(US Food and Drug Administration, 2020)
**Eptifibatide (Integrilin^®^) ^(2)^**	Pigmy rattlesnake (*Sistrurus miliarius*)	Synthetic	I.v. bolus injection (20 mg/10 ml); i.v. infusion (75 mg/100 ml); i.v. infusion (200 mg/100 ml); Each vial of any dose also contains 5.25 mg/ml citric acid and NaOH to adjust to pH 5.35.	Prevents binding of fibrinogen, von Willebrand factor, and other adhesive ligands to GPIIb/IIIa	Acute coronary syndrome; percutaneous coronary intervention	Initial dose of 180 µg/kg intravenous bolus administered and for maintenance 2 µg/kg/min by a continuous infusion until hospital discharge, or for up 18 to 24 h, whichever comes first. A minimum of 12 h of infusion is recommended by the manufacturer.	Bleeding, dizziness; US FDA pregnancy category B; not known if distributed into human milk	(RxList, 2019; European Medicines Agency, 2020)
**Exenatide (Byetta^®^) ^(2)^**	Gila monster lizard (*Heloderma suspectum*)	Synthetic	Prefilled cartridge pen (250 µg/ml; s.c. injection)	Glucagon-like peptide-1 receptor agonist	Type 2 diabetes mellitus	5 or 10 µg twice daily 60 min before two main meals of the day, ~6 h apart	Hypoglycemia, nausea, vomiting, diarrhea, jittery feeling, dizziness, headache, dyspepsia, asthenia, gastroesophageal reflux disease, hyperhidrosis, constipation, abdominal distention, decreased appetite, flatulence; data lacking on the use in pregnancy; not known if excreted into human milk	(US Food and Drug Administration, 2020)
**Extended-release exenatide (Bydureon^®^) ^(2)^**	Gila monster lizard (*Heloderma suspectum*)	Synthetic	Exenatide (2 mg) and diluent	Glucagon-like peptide-1 receptor agonist	Type 2 diabetes mellitus	2 mg weekly at any time of the dosing day, with or without meals	Hypoglycemia, nausea, diarrhea, injection-site reactions (pruritus, nodule, erythema, hematoma), vomiting, constipation, headache, viral gastroenteritis, gastroesophageal reflux disease, dyspepsia, fatigue, decreased appetite; data lacking on the use in pregnancy; not known if excreted into human milk	(European Medicines Agency, 2020; US Food and Drug Administration, 2020)
**Leech therapy**	European medicinal leech (*Hirudo medicinalis*) or other species	Leech	Leeches drain blood from tissue	Inhibits platelet aggregation and the coagulation cascade	Skin grafts and reattachment surgery	Usually 1–10 leeches are used for each treatment, while at the beginning, the patient might need two or more treatments per day. Leeches should be applied on the darker spots of the reattached body parts or flaps. Usually the treatment lasts for 2–6 days.	Lymphadenitis, slight swelling, pain of regional lymph nodes on the side of leech application and subfebrile temperature. Leech therapy is not recommended in pregnancy, lactation and in patients with an unstable medical status and disposition to keloid scar formation.	(Mumcuoglu, 2014; US Food and Drug Administration, 2020)
**Lixisenatide (Lyxumia^®^ and Adlyxin^®^) ^(2)^**	Gila monster lizard (*Heloderma suspectum*)	Synthetic	0.15 mg/3 ml (0.05 mg/ml)—s.c. 0.3 mg/3 ml (0.1 mg/ml)—s.c.	Glucagon-like peptide-1 receptor agonist	Type 2 diabetes mellitus	Initial dose: 10 µg by s.c. injection once a day. Increase to 20 µg on day 15. This drug should be administered 1 h before the first meal of the day. Concurrent use with short acting insulin has not been studied and is not recommended.	Nausea, vomiting, diarrhea, headache, dizziness, low blood sugar; data lacking on the use in pregnancy; not known if distributed into human milk, but its use is not recommended.	(European Medicines Agency, 2020; US Food and Drug Administration, 2020)
**Tirofiban (Aggrastat^®^) ^(3)^**	Saw-scaled viper snake (*Echis carinatus*)	Synthetic	I.v. bolus (3.75 mg in 15 ml—vial); i.v. bolus and infusion (5 mg in 100 ml—vial); i.v. bolus and infusion (12.5 mg/250 ml—bag)	Antagonist of fibrinogen binding to the GPlIb/lIla receptor	Acute coronary syndrome	Initial dose: 25 µg/kg i.v. within 5 min. Maintenance dose: 0.15 µg/kg/min i.v. infusion for up to 18 h	Dizziness, slow heart rate, leg pain, pelvic pain, swelling, increased sweating; US FDA pregnancy category B; not known if distributed into human milk.	(Medicure Pharma, 2016)
**Ziconotide (Prialt^®^) ^(2)^**	Magical cone marine snail (*Conus magus*)	Synthetic	25 or 100 µg/ml (aqueous pH adjusted solution pH 4-5, L-methionine and NaCl); i.t.	Cav2.2 channel antagonist	Severe chronic pain	Initial dose: ≤ 2.4 µg/day (≤ 0.1 µg/h) less than 2 to 3 times/week. Maximum dose: 19.2 µg/day (0.8 µg/h) by day 21.	Dizziness, confusion, drowsiness,abnormal gait, memory impairment, ataxia, speech disorder, headache, aphasia, hallucination, thinking abnormality, amnesia, anxiety, blurred vision, increased creatine phosphokinase, anorexia, nystagmus, fever; pregnancy risk factor C	(US Food and Drug Administration, 2020)

*For complete and detailed information, we suggest consulting Drugs.com and the patient information leaflets provided by the medicine manufacturer; 1), enzyme; 2), peptide; 3), non-protein molecule; i.t., intrathecal; i.v., intravenous; s.c., subcutaneous.

Most toxin-based approved drugs are derived from snake venoms. One of the possible reasons for this scenario is the larger amount of venoms produced by snakes in comparison to small animals (e.g. scorpions, spiders, and snails) (King, 2011; King, 2013). In parallel, the effect of snake venoms on hemostasis evidenced the cardiovascular system as a pharmacological target for snake venom toxins. Furthermore, the analytical techniques capable of characterizing limited amounts of venom components from small animals were only developed recently. These are some issues that boosted the initial toxinological studies primarly on snake venoms. The advent of more sensitive techniques and the improvement in experimental models in the last years have allowed the study of poorly expressed toxins and their novel pharmacological targets (Boldrini-França et al., 2017). Additionally, the discovery of many ion channels in the 1970-1980s, and the better understanding of the nervous system, which houses the main molecular targets of small venomous invertebrates, opened up the field to new therapeutic leads for non-cardiovascular targets (King, 2013).

The first animal toxin-based drug approved for human use was captopril in 1981. Captopril (Capoten^®^, Bristol-Myers Squibb) was developed based on the bradykinin potentiating factor (BPF) present in *Bothrops jararaca* snake venom (Ferreira, 1965; Camargo et al., 2012). BPF is a nonapeptide that acts by blocking the activity of the angiotensin-converting enzyme (ACE), inhibiting the production of the hypertensive molecule angiotensin II and potentiating the action of the hypotensive peptide bradykinin (Ferreira, 1965; Ferreira and Rocha e Silva, 1965; Ferreira et al., 1970a; Ferreira et al., 1970b).

Since the native peptide found in this venom was quite expensive to be synthesized and impossible to be orally administered (Ferreira, 2000), captopril was designed by the miniaturization of the original molecule, and by the addition of a succinyl group to a proline residue, which allowed its oral administration. This amino acid residue located at the C-terminal of BPP5a (one of the most active peptides in the bradykinin potentiating factor) is responsible for interacting with ACE (Cushman et al., 1977; Camargo et al., 2012). Captopril (alone or in combination with other drugs) is suitable and widely used for hypertension treatment (Weber et al., 2014).

After captopril, enalapril (MK-421, enalapril maleate) was approved by the FDA in 1985 for hypertension and congestive heart failure treatments (Patchett, 1984). The mercapto group in captopril structure was believed to be responsible for the skin rash and loss of taste reported as common adverse effects when using this drug. Therefore, the main challenge in enalapril development was to substitute the mercapto by an alkyl group, keeping the interaction with ACE (Patchett, 1984). Enalapril (Vasotec^®^, Merck) is produced as a prodrug that undergoes *in vivo* de-esterification to give rise to enalaprilat (MK-422), whose potency is greater than captopril, but has limited oral bioavailability (Biollaz et al., 1981; Patchett, 1984). Historically, captopril and enalapril are the hallmark in the development of ACE inhibitors for the treatment of hypertension.

The antiplatelet drug tirofiban (Aggrastat^®^, Medicure International, Inc.) is based on the RGD motif (Arg-Gly-Asp) from echistatin, a disintegrin found in the venom of the saw-scaled viper *Echis carinatus* (Topol et al., 1999). Tirofiban was approved by the FDA in 1998 for acute coronary syndrome treatment (Hartman et al., 1992). It mimics the RGD sequence and possesses a (S)-NHSO_2_-C_4_H_9_ group that enhanced the interactions with the platelet glycoprotein GPIIb/IIIa receptor (Gan et al., 1988). The competition with fibrinogen for the RGD recognition sites on the GPIIb/IIIa complex results in the inhibition of platelet aggregation and other antithrombotic properties (Topol et al., 1999; Lang et al., 2012).

Eptifibatide (Integrilin^®^, Millennium Pharmaceuticals, Inc.) is another antiplatelet drug approved by the FDA in 1998 and licensed to Schering-Plough in 2005. It was developed during the efforts to create synthetic analogues of barbourin, a disintegrin isolated from *Sistrurus miliarius barbouri* snake venom. Due to its conservative amino acid substitution of arginine (R) for lysine (K), barbourin presents more specificity for platelet glycoprotein GPIIb/IIIa complex than other disintegrins containing the RGD motif (Scarborough et al., 1991). Also, it was verified that the affinity for GPIIb/IIIa is highly influenced by the amino acid residues adjacent to the KGD sequence and the size of the peptide ring created through the disulfide bond formation. From this information, different synthetic peptides with potential clinical use were designed, including eptifibatide (Scarborough, 1999). Eptifibatide is a cyclic heptapeptide (deamino-Cys(1)-hArg-Gly-Asp-Trp-Pro-Cys(1)-NH_2_) more resistant to proteolysis due the introduction of a ring in the structure (Scarborough et al., 1993; Tcheng and O’Shea, 2002).

In the middle of 1900s, hirudin was isolated from *Hirudo medicinalis* leech saliva (Lee and Ansell, 2011). This 65-amino acid peptide presents an anticoagulant effect, through direct thrombin inhibition, and it was the only molecule to prevent blood coagulation until the discovery of heparin (Dodt et al., 1984; Markwardt, 1991). The removal of a sulfate group at Tyr63 residue gave rise to desulfatohirudin and increased in 10 times the complex formation with thrombin; however, obtaining this molecule with high activity and yield was a challenge to be overcome to allow clinical studies (Johnson et al., 1989; Markwardt, 1991). Desirudin (Iprivask^®^, Bausch Health), the recombinant 63-desulfohirudin (variant HV-1) produced in *Saccharomyces cerevisiae* (strain TR 1456), was approved by the FDA in 2003 for prophylaxis of deep vein thrombosis after hip replacement surgery (Warkentin, 2004). Revasc^®^ (Novartis) was approved by EMA in 1997, but it was withdrawn from the market in 2014 for commercial reasons.

In general, hirudins inactivate irreversibly thrombin, causing more bleeding than heparin (Römisch et al., 1993). Therefore, some analogues were developed with the aim of optimizing the therapeutic profile of hirudin based on the interaction with the active site of thrombin (Warkentin, 2004). Bivalirudin (Angiomax^®^, The Medicines Company) is a synthetic peptide resulted from rational drug design, comprised of 20 amino acids: 4 N-terminal residues from native hirudin which interact with the active site, connected by 4 glycine residues to the last 12 residues present in its C-terminal responsible to interact with the anion exosite (Maraganore et al., 1990). This drug binds reversibly to thrombin, which decreased the risk of bleeding reported to other hirudins (Nutescu and Wittkowsky, 2004). Angiomax^®^ was approved by the FDA in 2000 to patients with unstable angina undergoing percutaneous transluminal coronary angioplasty (Bittl et al., 2001). In Europe it is marketed as Angiox^®^ (Mehrzad et al., 2017).

In 2004, ziconotide (Prialt^®^, Elan Pharmaceuticals, Inc.) was approved for the management of severe chronic pain by the FDA and by the EMA (Smith and Deer, 2009). Ziconotide (SNX-111) is a synthetic analogue of the omega-conotoxin MVIIA isolated from the venom of the fish-hunting snail *Conus magus*. It is a 25-amino acid peptide that blocks Ca_v_2.2 channels (N-type voltage-sensitive calcium channels) and, consequently, inhibits the conduction of nerve impulse and release of neurotransmitters into the thalamus, leading to antinociception (McGivern, 2007; Vink and Alewood, 2012). Ziconotide does not induce dependence or tolerance, which is a valuable advantage in comparison to morphine, which is also less effective than ziconotide (Scott et al., 2002). However, the main limitations of ziconotide use are its intrathecal administration route, which impairs the patient’s adherence to the treatment (Smith and Deer, 2009), and narrow therapeutic index (Scott et al., 2002). Recently, the intranasal route has been studied to overcome the challenge of administering ziconotide (Manda et al., 2016).

Exenatide (synthetic exendin-4 from Gila monster, *Heloderma suspectum*) is the first glucagon-like-peptide-1 (GLP-1) analogue (Furman, 2012) and has been used as an adjuvant in the treatment of type 2 diabetes mellitus (Nauck et al., 2007; Henry et al., 2014). It presents a combination of actions: stimulation of insulin and suppression of glucagon secretion that result in blood glucose control, and reduction of body weight and cardiovascular risk factors (Eng et al., 1992; Greig et al., 1999; Alves et al., 2017).

Since GLP-1 is rapidly degraded by serum proteases presenting a very short lifetime, the key point was the development of GLP-1analogues resistant to these enzymes (Lorenz et al., 2013). Indeed the N-terminal (HGE) of exendin-4 is more resistant to peptidases that degrade the endogenous GLP-1 which make that more potent and longer-lasting than GLP-1 (Eng et al., 1992; Greig et al., 1999). The first pharmaceutical form of exenatide (Byetta^®^) was approved by the FDA in 2005 and in 2009 by the EMA. Even with the N-terminal more resistant to proteases, Byetta^®^ has a half-life of ~2.4 h after administration (Lorenz et al., 2013).

Lixisenatide (Lyxumia^®^ in the Europe and Adlyxin^®^ in the USA., Sanofi S.A.) is a 44-amino acid peptide, with an amide group on its C-terminus. It is comprised of the first 39 amino acids of exendin-4, with a deletion of proline at position 38 and addition of six lysine residues (Christensen et al., 2009). Lixisenatide was approved in 2013 and 2016 by the EMA and the FDA, respectively, as the first once-daily injectable GLP-1 receptor agonist for the treatment of diabetes type II, presenting a half-life of ~3 h (Elkinson and Keating, 2013; Lorenz et al., 2013; US Food and Drug Administration, 2016).

In addition, there is an extended-release form of exenatide (Bydureon^®^), approved in 2011 and 2012 by the EMA and the FDA, respectively. It has a half-life of 5-6 days due to its encapsulation into poly (D,L-lactide-*co*-glycolide) microspheres, which hydrate *in situ* and slowly degrade to release the drug over time, resulting in less peak-trough variation (DeYoung et al., 2011; Lorenz et al., 2013). Long-acting exenatide has also been developed in a ready-to-use auto injector to facilitate the administration since the former pharmaceutical form needs to be diluted prior to administration (Wysham et al., 2017).

Apart from the marketed drugs in the USA and Europe, there are also those approved by the National Medical Products Administration (NMPA, formerly State FDA and China FDA—SFDA and CFDA, respectively). Batroxobin (also known as hemocoagulase, reptilase, and botropase) is a thrombin-like serine protease obtained from *B. atrox* and *B. moojeni* snake venoms (Itoh et al., 1987; Earps and Shoolingin-Jordan, 1998). It cleaves fibrinogen, resulting in the formation of non-cross-linked fibrin clots. Unlike thrombin, which releases fibrinopeptides A and B from fibrinogen, batroxobin releases only fibrinopeptide A (Holleman and Weiss, 1976). Although the enzyme is not clinically approved in the USA, its defibrinogenating effect is clinically used in other countries for the treatment of various thrombotic diseases including deep vein thrombosis, myocardial infarction, pulmonary embolism, and acute ischemic stroke (You et al., 2004).

Currently, batroxobin has been commercialized with the brand names: Batroxobin and Reptilase (Tobishi Pharmaceutical, China), Defibrase (Tobishi Pharmaceutical, China and DSM Nutritional Products Ltd Branch Pentapharm, Switzerland), Botropase (Hanlim, South Korea and Juggat Pharma, India), Botroclot (Juggat Pharma, India) (Drugs.com, 2020), Plateltex-Act^®^ (Plateltex S.R.O., Czech Republic) (Plateltex, 2018), and Vivostat System (Vivostat A/S, 2018). Therapeutic applications of Defibrase^®^ include acute cerebral infarction, unspecific angina pectoris, and sudden deafness (Pentapharm DSM Nutritional Products Ltd, 2018). Plateltex-Act^®^ is used to prepare autologous platelet-gel, an emerging biotechnology in current tissue engineering and cellular therapy (Mazzucco et al., 2008). Batroxobin from Plateltex-Act^®^ converts fibrinogen into fibrin in the presence of Ca^2+^ ions, and forms a fibrin reticulum that causes the gelling of the product and cooperates with the regenerative and reparative processes of damaged tissues (Mazzucco et al., 2008; Plateltex, 2016). The Vivostat System (Vivostat A/S, Denmark) is a medical device used for the preparation of an autologous fibrin sealant in the operating room by the action of batroxobin upon the fibrinogen in the patient’s plasma (Vivostat A/S, 2018).

In 1998, cobratide (a short-chain post-synaptic α-neurotoxin isolated from *Naja naja atra* snake venom, also known as ketongning and cobrotoxin) was approved in combination with synthetic drugs as a pain killer for the treatment of moderate to severe pain (Gazerani and Cairns, 2014; Zhang, 2015). However, pharmacokinetics studies *in vivo* of cobratide injection (China Approval no. H53022101) are still necessary to adjust drug plasma concentrations and to reduce the risk of drug accumulation and fatal side effects (e.g., respiration inhibition) (Chen et al., 2016).

A detailed description of mechanism, pharmacology, pharmacokinetics, and clinical development of most approved toxin-based drugs can be found in specific reviews already published for each compound (Brogden et al., 1988; Tabacova and Kimmel, 2001; Wong, 2005; Zeymer, 2007; Graetz et al., 2011; Pope and Deer, 2013; Serrano, 2013; Knop et al., 2017; Trujillo and Goldman, 2017; Yang et al., 2019).

### Diagnostic Tools

Besides its therapeutic applications, batroxobin (Reptilase^®^) has also been used for decades as a laboratory reagent to measure fibrinogen levels and blood coagulation capability through the *in vitro* clotting time using serine proteases instead of thrombin (Reptilase^®^ time) (Funk et al., 1971). Since Reptilase^®^ does not need Ca^2+^ and phospholipids, some coagulation factors (V, VIII, XI, and XIII) are not activated and the platelet aggregation is not induced, cleaving only the fibrinopeptide A. Both Reptilase^®^ time and thrombin time are complementary tests to evaluate coagulation disorders. Reptilase^®^ is also used to detect antithrombin activity (Francischetti and Gil, 2019).

RVV-V (Pefakit^®^) is a 27 kDa factor V-activating serine protease from the Russel’s viper (*Daboia russelii*) venom, used to identify factor V levels in plasma (Tokunaga et al., 1988). It is widely used in assays for the diagnosis of resistance to activated protein C, which does not cleaves factors Va and VIIIa (Francischetti and Gil, 2019).

RVV-X (Stypven^®^) is a 120 kDa factor X-activating metalloprotease from *D. russelii* venom that converts factor X quantitatively into factor Xa (Tans and Rosing, 2001; Morita, 2005). This toxin is dependent of Ca^2+^, factor V, phospholipids and prothrombin (Francischetti and Gil, 2019).

Ecarin, from *E. carinatus* venom, is a 55 kDa metalloprotease able to activate prothrombin and detect its abnormal types (Morita et al., 1976; Weinger et al., 1980; Braud et al., 2000). Contrary to RVV-X, ecarin is independent of factor V, phospholipids or Ca^2+^, detecting thrombin with chromogenic substrates (Ecarin chromogenic assay—ECA) or in a clotting assay (Ecarin clotting time—ECT) (Francischetti and Gil, 2019).

RVV-V, RVV-X, and ecarin are used to the diagnosis of lupus anticoagulant (Francischetti and Gil, 2019), one of the clinical manifestations of Antiphospholipid Syndrome, characterized by the presence of antiphospholipid antibodies (Favaloro and Wong, 2014).

Other snake venom toxins used as diagnostic tools include Botrocetin^®^ and Protac^®^. Venom coagglutinin (Botrocetin^®^) isolated from *B. jararaca* venom is a 22 kDa C-type lectin-like protein that aggregates platelets by increasing the affinity between the receptor GPIbα and von Willebrand factor (Brinkhous et al., 1983; Beeton, 2013), independent of von Willebrand factor molecule size (Francischetti and Gil, 2019).

ACC-C (Protac^®^) from *A. contortrix contortrix* venom is a plasma protein C-activating serine protease used to quantify protein S and C levels (Stocker et al., 1988) with chromogenic substrates or by prolongation of the activated partial thromboplastin time (aPTT). These protein levels are used to investigate the cause of a blood clot (thromboembolism), linked to deep vein thrombosis or pulmonary embolism. AAC-C activity is not compromised by the inhibitor of protein C from plasma (Francischetti and Gil, 2019).

### Cosmeceuticals

The cosmeceutical field is a profitable venture. For example, the anti-wrinkling effect of the botulinum toxin (Botox^®^), a toxin isolated from *Clostridium botulinum* bacteria, accounts for striking global sales of about $3 billion per year (Clark et al., 2019). Among the biologically active compounds from animal venoms showing cosmeceuticals applications, we can cite the use of bee venom-containing cosmetics on facial wrinkles in human skin (Han et al., 2015), and the inhibitory activity of melanogenesis of Argiotoxine-636 (ArgTX-636), a polyamine isolated from *Argiope lobata* spider venom (Verdoni et al., 2016), including a deposited patent (US10064814B2) for skin whitening/depigmenting (Mabrouk et al., 2018). Another example is the synthetic tripeptide [dipeptide diaminobutyroyl benzylamide diacetate (H-β-Ala-Pro-Dab-NHBzl x 2 AcOH)], commercialized as the cosmeceutical SYN^®^-AKE (Pentapharm). It mimics the activity of waglerin 1, a 22-amino acid peptide from *Tropidolaemus wagleri* snake venom, and reduces wrinkles by inhibiting muscle contractions (Zhang and Falla, 2009).

### Venom Therapies

Bee venom therapy is an ancient therapy which uses this toxin arsenal as a cream, liniment, ointment, injection, acupuncture, or directly *via* stings of live bees to treat several disorders (Ali, 2012). Those treatments rely on the fact that bee venom is composed of a wide range of components, such as biogenic amines, enzymes (mostly PLA_2_s), basic peptides, and non-enzyme proteins (mainly melittin and apamin) (Santos et al., 2011). Bee venom acupuncture corresponds to the most common used method, especially in the Koreas, and can be employed as an alternative treatment to pain, rheumatoid arthritis, osteoarthritis, and multiple sclerosis. The treatment consists of using bee venom in the relevant sites according to the disease or acupuncture points (Lee et al., 2014). A phase II randomized study to evaluate the effects of bee venom acupuncture in 68 participants with adhesive capsulitis (frozen shoulder) (NCT01526031) and another one in 60 patients with chronic cervicalgia (NCT01922466) were completed in 2012 and 2015, respectively.

Bee venom designated as apitoxin (Apitox^®^) has been marketed by Apimeds, Inc. for osteoarthritis in South Korea since 2016. A phase III randomized study (NCT01112722) in 363 patients with diagnosed osteoarthritis of the knee was completed in 2016 and a phase III randomized study (NCT03710655) for multiple sclerosis is not yet recruiting patients (last update 2018). Apitox^®^ diminishes the pain and swelling associated with rheumatoid arthritis, tendinitis, bursitis, and multiple sclerosis (Bastos et al., 2011; Moreno and Giralt, 2015).

Another therapy for medicinal purposes is the hirudotherapy (medicinal leech therapy), approved in 2004 by the FDA. Since the beginning of civilization, leeches have been used for therapeutic purposes (Koh and Kini, 2008; Abdualkader et al., 2013). They are hematophagous animals that possess about 100 biologically active compounds in their saliva, especially the anticoagulants, but also components with anti-inflammatory, bacteriostatic, and analgesic properties (Singh, 2010). Many of the compounds responsible for those activities have already been identified (Sig et al., 2017), such as hirudin, kallikrein inhibitors, calin, hyaluronidase, collagenase, histamine-like substances, and antimicrobial peptides (e. g. theromacin, theromyzin, peptide B and lumbricin) (Cooper and Mologne, 2017). *H. medicinalis*, also known as the healing leech, is the main species used in the therapy (Abdualkader et al., 2013). The hirudotherapy has been shown to produce statistically significant improvement of arthritic conditions (Cooper and Mologne, 2017) and has also been applied in cardiovascular diseases, reconstructive and microsurgery, cancer and metastasis, diabetes mellitus and its complications, infectious diseases, arthritis, and as analgesic (Singh, 2010; Abdualkader et al., 2013). For an extensive review regarding these venom therapies, please see (Mumcuoglu, 2014; Jagua-Gualdrón et al., 2020).

**Figure 1:**
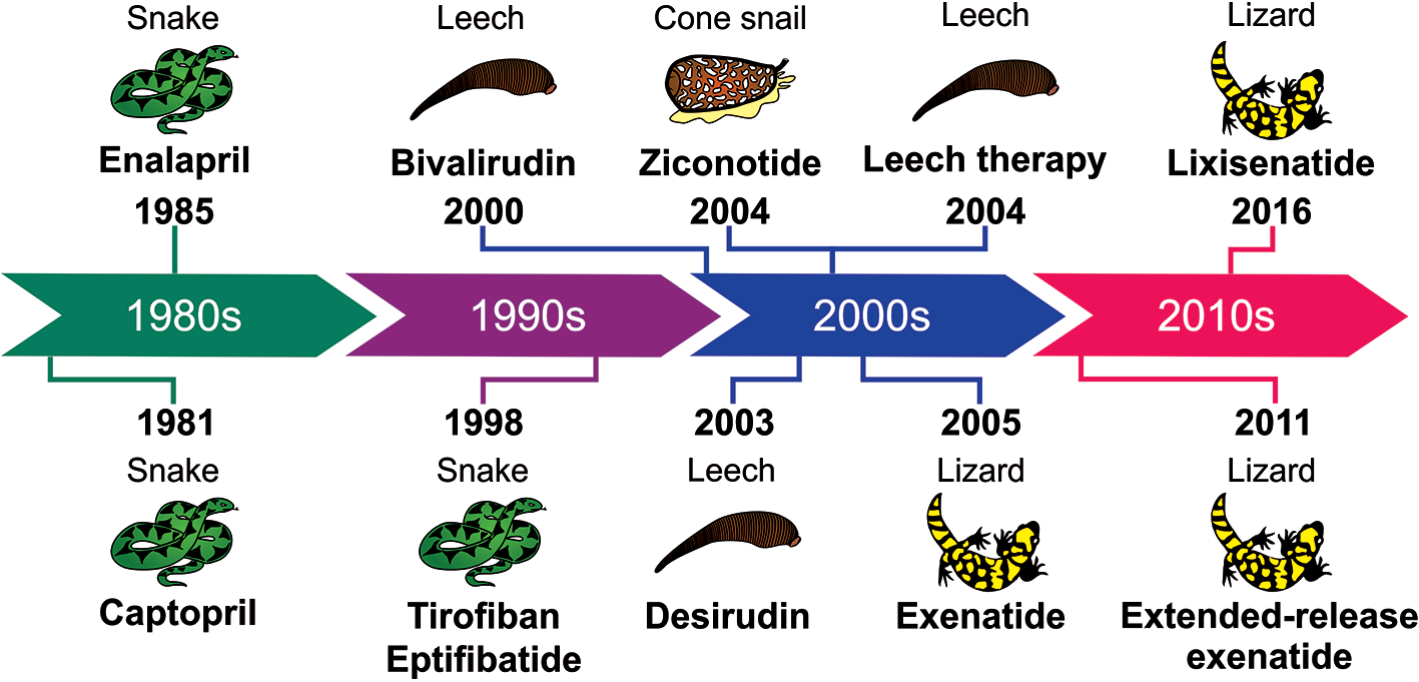
Timeline showing the animal toxin-based drugs and hirudotherapy approved by the FDA.

The hirudotherapy and all the toxin-based drugs approved by the FDA are chronologically shown in the next timeline (**Figure 1**).

## Animal Toxin-Based Drug Development Challenges

Animal toxins are most often useful as pharmacological tools for target validation. However, in section *Achievements With Animal Toxin-Based Molecules* it was shown that they have also been successfully used as therapeutic agents.

Although there are examples of success, there is a gap between the number of compounds with interesting pharmacological properties obtained from animal poisons and venoms and those that are approved. Drug development programs may be discontinued due to several factors, like intellectual property disputes, changes in the program leadership, lack of funding, among other business decisions. The lack of publications regarding important data, during the different stages of their development, also contributes to several program discontinuations. While we sought to retrieve this information from the scientific literature, this fact impairs most of the process, concealing most of the key events.

The subsections *Challenges Regarding Basic Research* to *Challenges Regarding Clinical Trials* will address the challenges related to basic research, preclinical evaluation and clinical trials during the development of animal toxin-based drugs. However, many challenges faced during these stages are not available in the scientific literature, since much of this information is under intellectual property law for compounds that are still being developed or for which the development stopped because of internal issues.

### Challenges Regarding Basic Research

One of the bottlenecks when studying toxins from small or rare venomous species, such as scorpions and spiders, is the hardship in obtaining large amounts of venom and purified toxins. For example, the venom glands from *Cupiennius salei* spider contain only 10 μl of venom, and the venom regeneration in milked animals requires from 8 to 16 days (Wigger et al., 2002). On the other hand, the snake *Lachesis muta muta* is able of injecting large venom amounts (milliliters of venom yielding 200-400 mg of toxins) (Stransky et al., 2018). The higher amount of collected snake venom is one of the reasons that may explain why most of the approved animal toxins-based drugs come from these animals.

Mucus-rich samples, such as toad and frog poisons, is also another issue, which may hinder the use of omic approaches (Shibao et al., 2018). In this context, studies comprising animal toxins are not a simple task since many challenges must be addressed. The small amount obtained from different poisonous and venomous animals, together with the nature of the venom/poison allied with the difficulty in isolating specific toxins, are the main limitations faced during basic research. Overcoming these limitations is thoroughly discussed in section *Filling the Gap Between the Drug Discovery and Its Commercialization—Future Trends*.

### Challenges Regarding Preclinical Evaluation

Problems in the development of toxin-based drugs encompass selectivity, mechanism of action, formulation, stability, and production cost (Zhang and Falla, 2009). Besides the modern approaches using omic techniques, molecular biology, bioconjugation, and nanomaterials in animal venom research, venom components do not always meet all the requirements for a potential therapeutic application. Drug metabolism and pharmacokinetics properties of animal toxins, for instance, are key factors that need to be carefully optimized (Kovalainen et al., 2015).

In this regard, after overcoming the challenges imposed during the basic research, like obtaining enough amount of the toxin, it becomes necessary to stand up against some pitfalls faced during preclinical evaluation. Some compounds lack the ability of crossing pivotal barriers in the organism, including the blood-brain barrier, which may interfere in their delivery. Additionally, the susceptibility to blood proteases, as well as their immunogenicity, which are directly linked to biopharmaceutical degradation *in vivo*, are also important factors to be considered. Due to the relatively large size and other specific physicochemical properties, parenteral administration is currently the most used delivery route for approved venom-based drugs (**Table 1**) (Ibraheem et al., 2014; Duskey et al., 2017).

Considering all the challenges at this phase, preclinical studies are usually costly and lengthy, since they must attend all the requirements stated by the regulatory agencies throughout the world. In this respect, regulatory issues, together with problems related to lack of funding, and manufacturing problems, have been a hindrance for academics pursuing to advance their drug candidates into the clinical trials.

### Challenges Regarding Clinical Trials

Randomized clinical trials are the gold standard to evaluate specific drug-related issues such as the efficacy and, to a lesser extent, the safety of new medicines before marketing approval. But these studies are not often able to evaluate special populations, such as children, pregnant women, and the elderly (Trifiro et al., 2019). To overcome these limitations, studies using electronic healthcare records (EHRs) of post-marketing comparative drug safety may complement traditional spontaneous reporting systems to predict which drugs require further epidemiological investigation. For instance, a multi-country healthcare database network identified new signals of potentially drug-induced acute liver injury in children using EHRs (Ferrajolo et al., 2014). A method of enhancing effectiveness of therapeutic agents using taxane nanoparticle co-administered with the therapeutic agent has been recently patented (US10660965B2).

On this point, the obstacles faced during the process of approving a new drug are harder to overcome than just improving its drugability, with two mainly issues contributing at this stage. First, new therapeutic drugs must achieve very high standards to be accepted, since they may have to compete with older and well-known drugs on the market, which may be more effective and cheaper, in most cases (because of the expired patent, for instance) (Scannell et al., 2012). Another problem is when the role of the toxin’s target on the disease state is less relevant than previously thought for the manifestation of a particular disease, resulting in low efficacy. Even more, unexpected and unwanted effects could be observed *in vivo* if the target is expressed at different cells or if the toxin binds promiscuously to other targets (Scannell et al., 2012; Harvey, 2014; Vetter et al., 2017). In this context, adverse effects, lack of efficacy and dose-limiting toxicity are responsible for the interruption of many clinical trials (Harvey, 2014; Lewis, 2015).

## Learning From Discontinued Toxin-Based Drugs

Most cases of drugs withdrawn from the market (voluntarily or prohibited by regulatory agencies) are related to different events, ranging from safety issues, like serious side effects, to several non-safety issues, encompassing those related to the manufacturing process, regulatory or business issues, or lack of efficacy. The foreseen toxicity of some toxin-based drugs may not be completely avoided, impairing the process at different stages of drug development. Therefore, understanding the mechanisms of toxicity is of utmost importance as an attempt to prevent post-marketing withdrawals (Siramshetty et al., 2016).

A mimetic peptide isolated from *Naja* spp. cobra venom, ximelagatran (Exanta^®^, AstraZeneca), was discontinued in 2006, due to hepatotoxic potential (King, 2011). This prodrug anticoagulant agent, orally administered, had been approved in Europe and South America for thrombin inhibition (Eriksson et al., 2003; Koh et al., 2006; Fox and Serrano, 2007; King, 2011). While Ximelagatran was mostly well tolerated in specific trial populations, a small proportion of the treated patients developed elevated liver enzyme levels, during phase II of clinical trials, which caused the FDA to reject its approval.

A phase III study of agkisacutacin (also known as hemocoagulase) in perioperative bleeding (Wei et al., 2010) was ceased due to anaphylactic reactions (Xu et al., 2016). The enzyme, which acts on fibrinogen and fibrin, is a heterodimeric serine protease from *Deinagkistrodon acutus* venom whose monomers A and B are comprised of 123 and 129 amino acid residues, respectively, linked by a disulfide bond (Wei et al., 2010). On the other hand, a phase IV randomized study (NCT03270735) to evaluate the efficacy and safety of hemocoagulase injection in the treatment of moderate to severe hemoptysis is recruiting patients since 2017. However, updated information regarding the evolution of this study could not be retrieved.

Pexiganan, also known as MSI-78 (a 22-residue linear peptide analogue of magainin-2), isolated from the skin of *Xenopus laevis* frog, is an antimicrobial peptide with therapeutic potential in treatment of infected foot ulcers in diabetic patients. The molecule presents *in vitro* activity against both Gram-positive and Gram-negative bacteria. The company Dipexium Pharmaceuticals, Inc. patented a 0.8% pexiganan acetate cream (Locilex^®^ or Cytolex) but, in 1999, FDA denied the approval of this medicine arguing that its efficacy was not proven superior to that of the conventional treatment in any of the clinical trials (Ladram and Nicolas, 2016; Gomes et al., 2017).

Following the approval of ziconotide, other conotoxins, such as leconotide and Xen2174, were synthesized, studied and advanced to clinical trials. Leconotide (AM336 or ω-conotoxin CVID from *Conus catus*) caused side effects when intrathecally administered and would be intravenously evaluated, but the developer company went bankrupt (Harvey, 2014). Xen2174 (χ-CTX MrIA from *C. marmoreus*) progressed to Phase IIb trial (Lewis, 2015), but it showed dose-limiting toxicity in pharmacodynamics and cerebrospinal fluid pharmacokinetics assays. Thus, it is unlikely that this conotoxin can be used for the treatment of acute pain in humans (Okkerse et al., 2017).

Alfimeprase, a recombinant zinc metalloprotease fibrolase from *Agkistrodon contortrix* with 203 residues and three disulfide bonds, cleaves the Aα- and Bβ-chains of fibrin, releasing fibrinopeptides A and B, respectively (King, 2011; Koh and Kini, 2012; Swenson and Markland, 2013). This molecule reached phase III of clinical trials in catheter occlusion and stroke; however, it was discontinued due to the lack of effectiveness (Shah and Scher, 2007; Markland and Swenson, 2010).

Among the several reasons for the interruption of many drug development programs are also intellectual property conflicts, lack of funding, business issues or changes in development leadership. In the case of lepirudin (Refludan^®^), for instance, its marketing was discontinued by Bayer in 2012 because the third-party manufacturer of the product had permanently ceased production of the drug (Bayer Healthcare, 2012). But the reasons that led to the manufacturing interruption have not been published, which prevents the proposal of solutions. Lepirudin is a recombinant peptide similar to hirudin, with an isoleucine instead of a leucine at N-terminal region and also lacking a sulfate group at Tyr63. It was marketed for prophylaxis or treatment of thrombosis complicating heparin-induced thrombocytopenia (Lee and Ansell, 2011).

The process of looking for information on drug removals from the market or haltered developments is a difficult task, since some of them are not available for several reasons aforementioned, and the data retrieved from public databases are significantly limited. In other words, factors that have not been published could have contributed to the discontinuation of the program.

## Promising Animal Toxins in Preclinical Stage and Clinical Trials

The database search for toxin-based drugs on clinical trials is challenging. One needs to know the acronym or the abbreviation of the desired active ingredient, since sometimes neither the species nor the generic name is cited to allow a broad search. Furthermore, most of the information on these drugs is confidential and thus not available in the public domain. Another problem is that a lot of available data for some drugs have not been updated for several years, which makes it difficult to find accurate details.

Clinical development is a lengthy and costly process that includes phases I to III of clinical trials (previous regulatory review and approval) and phase IV (post-marketing surveillance) (Chow and Chang, 2008). Phase I recruits healthy volunteers to assess primarily pharmacokinetics, safety and tolerability; phase II evaluates a cohort of patients with the target disease to establish efficacy and dose-response relationship, and the large-scale phase III studies confirm safety and efficacy (Tamimi and Ellis, 2009). Phase IV clinical development focus on the safety rather than efficacy (Chow and Chang, 2008).

The following subsections will address the clinical trial status of some toxin-based drugs from different animal species and additional information about these drugs is available in **Table 2**.

**Table 2:** Toxin-based drugs in clinical trials.

Molecule (NCT number)	Species origin of venom toxin	Production	Formulation	Mechanism of action	Use	Status(last update)	Reference
**ACV-1 (α-Vc1.1) – Discontinued ^(1)^**	*Conus victoriae*	Synthetic	S.c. injection	Activation of GABA_B_ receptors	Neuropathic pain	Phase II—discontinued (lack of efficacy)	(Clark et al., 2010; King, 2011)
**Agkisacutacin, Hemocoagulase, Recothrom^®^ (NCT not available; NCT03270735) ^(2)^**	*Deinagkistrodon acutus*	Recombinant	I.v. infusion (2U)	Fibrinogen and fibrin cleavage	Perioperative bleeding; moderate to severe hemoptysis	Phase III ceased (2016); phase IV recruiting (September 1, 2017)	(Wei et al., 2010)
**Alfimeprase – Discontinued (NCT00338585) ^(2)^**	*Agkistrodon contortrix contortrix*	Recombinant	Parenteral administration (up to 0.5 mg/kg was tolerated)	Cleaves Aα-chain of fibrin and fibrinogen	Catheter occlusion and stroke	Phase III terminated (based upon preliminary safety and efficacy results from a similar study) (January 15, 2008);	(Swenson et al., 2004; Ouriel et al., 2005)
**Ancrod (Viprinex^®^) (NCT01621256) ^(2)^**	*Calloselasma rhodostoma* (Malasyan pit viper)	Recombinant	I.v. infusion (0.167 IU/kg for 6 h)	Reduce fibrinogen	Sudden sensorineural hearing loss	Phases I/II completed (December 21, 2018)	(Hennerici et al., 2006)
**Bombesin (NCT02440308) ^(1)^**	*Bombina bombina*	Synthetic	I.v.	Attaches to prostate tumor cells with specific receptors on their surfaces	Imaging agent for positron emission tomography/magnetic resonance imaging	Phase II completed (April 11, 2017)	(US National Library of Medicine, 2020)
**Cenderitide, CD-NP (NCT00482937, NCT02603614, NCT02359227 and NCT02071602) ^(1)^**	*Dendroaspis angusticeps*	Chimeric natriuretic peptide	I.v. infusion (10, 25, 50, 100, 200, and 300 ng/kg/min over 4 h) or subcutaneous infusion (0.5, 1.0, 2.0, and 3.0 ng/kg/min) or IV infusion (5 and 10 ng/kg/min over 72 h)	Connection to natriuretic peptide receptor	Congestive cardiac failure, heart failure and myocardial infarction	Phase I completed (June 6, 2007; February 11, 2020 and January 4, 2019)	(Lee et al., 2009)
**Chlorotoxin derivates - Tozuleristide (BLZ-100) (NCT02234297) and 131-I-TM-601 (NCT00379132) ^(1)^**	*Leiurus quinquestriatus quinquestriatus* (Deathstalker yellow scorpion)	BLZ-100: Synthetic/131-I-TM-601: Recombinant	I.v. infusion (BLZ-100: dose unknown/31-I-TM-601: 0.2, 0.4, and 0.6 mg)	Binds to different targets (membrane type-2 matrix metalloprotease, annexin A2, and CLC-3 chloride channels in glioma cells and other tumors of neuroectodermal origin)	Tumor paint for intraoperative visualization of solid cancer cells	Phase I completed (April 6, 2016 and March 31, 2009)	(Patil et al., 2019; US National Library of Medicine, 2020)
							
**Cinobufacini (Buformin^®^) (NCT02871869)**	*Bufo gargarizans* or *Duttaphrynus melanostictus*	Sterilized water extract of dried toad skin (Chansu)	0.3 g per tablet, three tablets per time	Induction of apoptosis; inhibition of cancer cells	Several types of cancer	Phase II/III recruiting (July 17, 2017)	(US National Library of Medicine, 2020)
**Conantokin-G (CGX-1007) – Discontinued ^(1)^**	*Conus geographus*	Synthetic	Intrathecal	NMDA receptor antagonist	Intractable epilepsy	Phase I—discontinued (the developer company went bankrupt)	(Han et al., 2008; King, 2011)
**Contulakin-G (CGX-1160) – Discontinued ^(1)^**	*Conus geographus*	Synthetic	Intrathecal	Neurotensin receptor agonist	Neuropathic pain	Phase II—discontinued (the developer company went bankrupt)	(Han et al., 2008; King, 2011)
**Dalazatide, ShK-186, Stichodactyla toxin ShK (NCT02435342) ^(1)^**	*Stichodactyla helianthus* (Sun sea anemone)	Synthetic	S.c. injection twice per week for a total of nine doses	Kv1.3 channel antagonist	Autoimmune diseases (psoriatic arthritis, multiple sclerosis, lupus, rheumatoid arthritis, etc.)	Phase I completed (May 6, 2015)	(US National Library of Medicine, 2020)
**Desmoteplase (NCT00790920 and NCT00111852) ^(2)^**	Common vampire bat (*Desmodus rotundus*)	Recombinant	I.v. single bolus (90 or 125 µg/kg of body weight)	Plasminogen activator a1 with high fibrin specificity	Acute ischemic stroke	Phase III completed (September 18, 2015 and March 20, 2012)	(US National Library of Medicine, 2020)
**Fibrin glue, fibrin sealant ^(2 and 3)^**	*Crotalus durissus terrificus* and *Bubalus bubalis*	Thrombin-like serine protease from snake venom and fibrinogen-rich cryoprecipitate from buffalo blood	Topically	Fibrinogen cleavage	Adhesive, sealant, and hemostatic effects	Phase I/II completed by the Clinic of Chronic Ulcers of the Dermatology Service at the Botucatu Medical School, UNESP (November, 11, 2019)	(Ferreira et al., 2017; Buchaim et al., 2019)
**Huachansu (NCT00837239 and NCT02647125)**	*Bufo gargarizans* or *Duttaphrynus melanostictus*	Sterilized water extract of dried toad skin (Chansu)	20 ml/m^2^ for total 500 ml given as a 2-h infusion	Induction of apoptosis; inhibition of cancer cells	Several types of cancer	Phase II completed (July 12, 2012) and active, not recruiting (April 30, 2020)	(US National Library of Medicine, 2020)
**Leconotide (AM336 or ω-conotoxin CVID) – Discontinued ^(1)^**	*Conus catus*	Synthetic	3–6 µg/h (intrathecal)	Selective blocker of Ca_V_2.2 channel	Neuropathic pain	Phase I/IIa—discontinued (the developer company went bankrupt)	(Kolosov et al., 2010; King, 2011; Harvey, 2014)
**RPI-78M (Receptin^®^) (NCT not avaiable) ^(3)^**	*Naja kaouthia*	Detoxified or chemically modified	Orally (with benzalkonium chloride)	Connetion to nicotinic acetylcholine receptors (nAChRs)	Analgesic applications and multiple sclerosis	Manufacturing for clinical trials (October 28, 2018)	(King, 2011; Drug discovery and development, 2016; Adis Insight, 2018; Ojeda et al., 2018)
**RPI-MN (Pepteron^®^) (NCT not avaiable)^(3)^**	*Naja naja atra*	Detoxified or chemically modified	S.c. injection	Connection to nicotinic acetylcholine receptors (nAChRs) and can protect cells due to its ability to inhibit viral replication	Analgesic applications and HIV	Phase I and II completed (January 28, 2020)	(Biocentury, 2007; King, 2011; Adis Insight, 2020)
**Shk-192 – Discontinued ^(1)^**	*Stichodactyla helianthus*	Synthetic	10 or 100 µg/kg by s.c. injection once daily	K_V_1.3 channel blocker	Autoimmune disease	Phase I	(Pennington et al., 2009; King, 2011)
**Soricidin, SOR-C13 (NCT01578564 and NCT03784677) ^(1)^**	*Blarina brevicauda* (Northern short-tailed shrew)	Synthetic	I.v. infusion (dose range from 1.375 to 6.12 mg/kg)	Inhibitor of the Ca^2+^-selective transient receptor potential channel TRPV6	Ovarian (and other) cancers	Phase I completed (June 23, 2016) and recruiting (August 6, 2019)	(US National Library of Medicine, 2020)
**Tetrodotoxin (Tectin^®^) (NCT01655823) ^(4)^**	*Pufferfish, marine animals and phylogenetically unrelated terrestrial organisms*	Synthetic	Different injectable dosages (1 ml), twice a day for four consecutive days	Sodium channel blocker	Neuropathic pain caused by chemotherapy	Phase II terminated (decided to proceed to Phase III) (October 30, 2018)	(US National Library of Medicine, 2020)
**Xen 2174 (χ-CTX MrIA) – Discontinued ^(1)^**	*Conus marmoreus*	Synthetic	Intrathecal	Interacts with a large hydrophobic pocket within the norepinephrine transporter	Postoperative pain	Phase IIb (2015)— discontinued	(Brust et al., 2009; King, 2011; Lewis, 2015)
**Ximelagatran (Exanta^®^) – Discontinued (NCT00206089) ^(4)^**	*Naja* spp.	Synthetic	36 mg orally twice daily.	Direct thrombin inhibitor	Prevention of venous thromboembolic events	Phase III terminated (November 15, 2010)—withdrawn from the market and clinical development in February 2006 in the interest of patient safety (hepatic toxicity)	(Gulseth, 2005; King, 2011)

1), peptide; 2), enzyme; 3), non-enzyme protein; 4), organic molecule; i.v., intravenous; NCT, ClinicalTrials.gov identifier; s.c., subcutaneous.

### Amphibians

Chansu, the dried toad venom secreted by the skin glands of *Bufo gargarizans* (previously *B. bufo gargarizans*) or *Duttaphrynus melanostictus* (previously *B. melanostictus*), has been used in the Traditional Chinese Medicine for more than 1000 years (Qi et al., 2011). Bufalin, the major digoxin-like immunoreactive component of Chansu, is a cardiotonic glycoside (bufadienolide) present in toad poisons and has demonstrated anticancer activities in several preclinical studies (Miao et al., 2013). Cinobufagin and resibufogenin are also bufadienolides present in Chansu, capable of inhibiting cancer cells growth *in vitro* (Xie et al., 2012; Li et al., 2013).

Huachansu (also known as cinobufacini) is a sterilized aqueous extract of Chansu, designed for intravenous injection, and has been widely used in oncological clinics in China to treat patients with several types of cancer, being approved by the NMPA (formerly China FDA) (Qi et al., 2011; Liu et al., 2015). The major biologically active components present in huachansu are steroidal cardiac glycosides, such as bufalin, resibufogenin, cinobufagin, cinobufotalin, marinobufagin (also known as marinobufagenin) and bufotalin, and indole alkaloids, like bufotenine, bufotenidine, cinobufotenine, and serotonin (Su et al., 2003).

During a phase I clinical study, huachansu was tolerable even at doses 6 times higher than those normally administered, and could slow disease progression in some cancer patients, with no observed significant cardiac toxicity (Meng et al., 2009).

The efficacy and safety of gemcitabine-oxaliplatin (Gemox) combined with huachansu chemotherapy is an effective and well-tolerated regimen for advanced and metastatic gallbladder carcinoma (Qin et al., 2008). Another study showed that huachansu combined with chemotherapy reduced the occurrence of gastrointestinal side effects and leukocytopenia in patients with advanced gastric cancer (Xie et al., 2013).

Many *in vitro* studies demonstrating anticancer properties of huachansu justify its continued evaluation in clinical trials. Phases II and III studies started recruiting participants, in 2016, to evaluate if cinobufacini tablets have synergistic effect in the treatment of diffuse large B cell lymphoma, the most common subtype of non-Hodgkin lymphoma. The estimated date of conclusion of the study is December 2021 (NCT02871869).

Bombesin is a peptide composed of 14 amino acids (EQRLGNQWAVGHLM-NH_2_), isolated from the poisonous skin of the frog *Bombina bombina*, that shows high affinity for gastrin-releasing peptide-receptors (Tornesello et al., 2017; Utkin, 2017). Overexpression of members of this receptor family has been documented in several human neoplasms, such as prostate cancer, breast cancer, and small cell lung cancer. In this way, these receptors represent a molecular target for radiolabeled bombesin analogues as diagnostic or radiotherapeutic applications in these tumors (Schwartsmann et al., 2006; Wieser et al., 2014). Gallium-68 (68Ga)-DOTA-bombesin completed phase II in 2017 with 10 patients presenting prostate cancer. It is an imaging agent for positron emission tomography/magnetic resonance imaging and attaches to tumor cells with specific receptors on their surfaces (NCT02440308).

Different chemical modifications have been introduced in the synthetic bombesin to stabilize its structure, increase the binding affinity and to potentiate its agonist/antagonist properties (Cescato et al., 2008; Tornesello et al., 2017). A large variety of bombesin receptor ligands have been preclinically tested, most of which were bombesin agonists (Baratto et al., 2018). However, most of these ligands demonstrated high gastrointestinal uptake and limited metabolic stability *in vivo*, and can cause acute side effects (nausea, abdominal pain and emesis) when administered at higher doses (Accardo et al., 2016).

A synthetic bombesin/gastrin-releasing peptide-receptor antagonist (RC-3095) was able to produce long-lasting tumor regressions in murine and human tumor models *in vitro* and *in vivo*. Due to the occurrence of local toxicity at the injection site during a phase I trial in patients with advanced solid malignancies, a recommended dose of RC-3095 for Phase II trials could not be clearly established (Schwartsmann et al., 2006). Considering its mechanism of action and preclinical antitumor activity, further studies exploiting new formulations of RC-3095 for human use, such as slow-release preparations and analogues with a more favorable pharmacokinetics, are justified.

Epibatidine is an alkaloid extracted from the skin of the Ecuadorian frog *Epipedobatus tricolor* (poison-dart frog). This molecule binds to several nAChR subtypes, including α7, α4β2, and the neuromuscular α1β1δγ subtype. Antinociceptive efficacy of epibatidine is about 100 times more powerful than morphine, but it has induced adverse effects, revealing high toxicity to be used as a pain-relieving drug (Traynor, 1998; Salehi et al., 2019).

Many compounds based on the chemical structure of epibatidine have been developed and tested to become new, powerful pain-reducing drugs (Daly, 2004; Umana et al., 2013). An example is ABT-594 (tebanicline or ebanicline) (Salehi et al., 2019). ABT-594 is of particular interest once it is more powerful than morphine showing no morphine-associated side effects and only mild cardiovascular side effects (Fox and Serrano, 2007). Due to severe gastrointestinal side effects caused by this first analogue of epibatidine, it has not been included in pain therapies in humans (Salehi et al., 2019).

### Cone Snails

Conotoxins, isolated from different species of cone snails (*Conus* ssp.), comprise a large family of small cysteine-rich peptides (10–30 amino acid residues) organized in subfamilies according to their structure (cysteine framework) and their mechanism of action (Lewis et al., 2012; Ovsepian et al., 2019). Undoubtedly, omega-conotoxins represent the most notable and famous conotoxin subfamily, in which omega-MVIIA [ziconotide (Prialt^®^)], previously reported in the section *Achievements With Animal Toxin-Based Molecules*, belongs to.

KCP-400 (also known as RgIA4), derived from Vc1.1, the first toxin isolated from *C. regius* venom, is a novel non-opioid drug for the treatment of chronic pain. Vc1.1 is a highly potent toxin that targets α9α10 nAChR, blocking pain signaling at the site of nerve injury, producing analgesic, anti-inflammatory and neuroprotective effects (Romero et al., 2017). The preclinical safety and efficacy studies of KCP-400 had been conducted by Kineta Inc., which is currently developing the non-opioid KCP-506 (Kineta Inc., 2020).

Because of their high potency and specificity, novel conotoxins can provide additional information on the pharmacology of ion channels, receptors, and transporters (Lewis et al., 2012; Gao et al., 2017).

### Hymenopterans

The whole venom of bees (Alyostal ST *Apis mellifera*) completed a randomized phase II study, in 2014, to evaluate its efficacy and potential effects in 50 participants presenting motor symptoms of Parkinson’s disease (NCT01341431). The administration of bee venom showed to be safe in non-allergic patients (Hartmann et al., 2016). Following the same direction of bee venom, the whole venom of ants has been employed in therapeutic use. For instance, the extracted material from venom sacs of *Pseudomyrmex triplarinus* could be helpful in relieving the pain caused by rheumatoid arthritis (WO1990003178A1, US4247540A).

The whole venom of wasps, bees and ants are also being used in venom immunotherapy (VIT), which represents a treatment to allergic patients preventing further sting-induced anaphylactic reactions (Kolaczek et al., 2017). Several clinical protocols and guidelines were published and generally consist of injections of small but gradually increasing doses of a specific venom (Bonifazi et al., 2005).

Despite those studies published employing hymenoptera whole venoms, little has been reported on the therapeutic applications of purified toxins. Thus far, the most explored hymenoptera venom components are melittin, apamin (both isolated from bees), and mastoparan (isolated from wasps) (Moreno and Giralt, 2015). All those three components arise as promising drug candidates for several conditions or therapeutic applications, such as antitumor agents (Gajski and Garaj-Vrhovac, 2013; de Azevedo et al., 2015), learning disabilities (Messier et al., 1991; Ikonen and Riekkinen, 1999), antimicrobial and antiviral activity (Vila-Farres et al., 2012; Sample et al., 2013), cell penetrating-peptides (Jones and Howl, 2012), among other applications.

Concerning melittin, a phase II study of ARC-520 in 79 participants with chronic hepatitis B virus (HBV) was terminated for regulatory and business reasons in 2019 (NCT02577029). The Dynamic Polyconjugate^®^ technology, developed by Arrowhead Therapeutics, uses melittin as an endosomolytic agent to facilitate the delivery of siRNA conjugates to hepatocytes (US8313772; US8501930; US8618277; WO2013003520A1).

### Scorpions

Chlorotoxin (CTx) is the only toxin from scorpion venoms undergoing clinical phase trials. The evidence of a venom molecule that interacts with chloride (Cl^-^) channels was firstly demonstrated by DeBin and Strichartz, which showed that *Leiurus quinquestriatus quinquestriatus* (the yellow scorpion from the Middle East, also known as death stalker) venom was able to block Cl^-^ channels of reconstituted rat epithelia and embryonic rat brain (DeBin and Strichartz, 1991). CTx is a peptide with 36 amino acids presenting 4070 Da, 4 disulfide bonds and it is positively charged in pH 7. Moreover its structure was solved by nuclear magnetic resonance spectroscopy: three-stranded antiparallel β-sheet packed against an α-helix (Lippens et al., 1995). The synthetic CTx was also produced successfully (Ojeda et al., 2016).

CTx discovery was marked by a substantial rise of publications using this molecule for different applications, such as insecticide (DeBin et al., 1993), antiangiogenic (Jacoby et al., 2010), and tumor binding (Cohen-Inbar and Zaaroor, 2016). CTx has demonstrated the capability to bind to different targets including chloride channels, membrane type-2 matrix metalloprotease (MMP-2) and annexin A2 (Ojeda et al., 2016). However, a milestone in the CTx discovery was the production of fluorescent molecular probes such as the tumor paint (CTx conjugated with Cy5.5 or CTx : Cy5.5). This bioconjugate can detect cancer foci and metastases from malignant glioma, sarcoma medulloblastoma and prostate and intestinal cancers using mouse models. The specific identification by this fluorescent molecular beacon (CTx : Cy5.5) increases the precision of surgical resection (image guidance) and improves patient prognosis (Veiseh et al., 2007). CTx:800CW (an infrared dye conjugate) was also produced; however, it has failed since the integrity of the blood-brain barrier was compromised even in the early stages of medulloblastoma tumor (Kovar et al., 2013).

Tozuleristide (BLZ-100), a CTx indocyanine green conjugate, demonstrated to bind to tumor cells while sparing healthy tissues (Butte et al., 2014). Phase I studies of BLZ-100 in 17 patients with glioma undergoing surgery were finished in 2016 (NCT02234297). The 131-I-TM-601 is the recombinant version of chlorotoxin (TM-601) radioconjugated with iodine 131 (Hockaday et al., 2005; Kesavan et al., 2010). It has been tested against different cancers (breast cancer, non-small cell lung cancer, melanoma, colorectal cancer, pancreatic cancer, prostate adenocarcinoma, glioma primary and solid tumors). The Phase I with 60 patients presenting recurrent or refractory somatic and/or cerebral metastatic solid tumors was completed in 2009 (NCT00379132). Regarding intellectual property, many patents applications can be detected relating to CTx variants, bioconjugates and methods for use, with an extensive list of records (e.g. WO2011142858A2; WO20006115633A2; US20030021810A1; US20160096869A1; US20080260639A1).

Although solely CTx reached clinical phase so far, other scorpion toxins have demonstrated therapeutic potential. For instance, the scorpion venom active polypeptide (SVAP) from *Mesobuthus martensii* (formerly *B. martensii*) has completed preclinical phase as a potential antithrombotic peptide. The results demonstrated that SVAP (0.125, 0.25, 0.5 mg/ml) inhibited rabbit platelet aggregation *in vitro*. Moreover, this peptide (0.32 and 0.64 mg/kg, intravenous administration) prolonged the occlusion time of carotid artery thrombosis in rats. Thus, SVAP may be considered an interesting molecule to be used in the treatment of cardiocerebral vascular diseases (Song et al., 2005).

Cancer treatment is also explored with other scorpion toxins. Besides CTx, BmKCT, a CTx-like molecule from *M. martensii* venom, reversibly inhibits chloride currents of glioma cells (Yang et al., 2005). BmkTa, also a CTx-like from *M. martensii* venom, is able to abolish the human glioma cells growth in a dose-dependent manner, with an IC_50_ of approximately 0.28 µM (Fu et al., 2007). Although some peptides must be highlighted (AmmTx3, BmTx3, Bekm-1, BmHyA, and IbTx), the list of scorpion toxins with antiproliferative activities is extensive (Das Gupta et al., 2007; Fu et al., 2012; Ding et al., 2014; Ortiz et al., 2015).

Scorpion toxins blocking potassium channels have also been widely investigated. In particular, those inhibiting K_v_1.3 currents are considered potential bioactive molecules to treat autoimmune diseases (Zhao et al., 2015). To the best of our knowledge, there are 81 scorpion toxins with positive results in inhibiting K_v_1.3 (Oliveira et al., 2019). Nevertheless, only eight of them present *in vivo* assays (i.e. most of them were studied using solely *in vitro* electrophysiological experiments). 1) HsTX1 from *Heterometrus spinifer* venom demonstrated to reduce inflammation in an active delayed-type hypersensitivity model and in the pristane-induced arthritis using rat models (Tanner et al., 2017). 2) ImKTX88 from *Isometrus maculatus* venom ameliorates pathological severity in rat experimental autoimmune encephalomyelitis (Huang et al., 2017). 3) Kaliotoxin (Ktx) from *Androctonus mauretanicus mauretanicus* venom showed the ability of preventing bone loss through a receptor activator of NF-κB ligand (RANKL)-dependent osteoclastogenesis mechanism, using rat periodontal disease model. Thus, Ktx has been tested to treat periodontal disease and rheumatoid arthritis (Valverde et al., 2004). 4) Margatoxin (MgTX) from *Centruroides margaritatus* venom caused a reduction of tumor volume into a xenograft model using nude mice by blocking K_v_1.3 channels, and it is being considered as a novel therapeutic target for lung adenocarcinoma therapy (Jang et al., 2011). 5) OSK1 from *Orthochirus scrobilosus* venom displayed blocking activity of K_v_1.3; however, during *in vivo* experiments, it demonstrated to be neurotoxic since it can diffuse immediately throughout the mouse brain (Mouhat et al., 2005). 6-7) Ts6 and Ts15 from *Tityus serrulatus* venom inhibit the proliferation of effector memory T cells and reduce inflammation in delayed-type hypersensitivity response using mice model (Pucca et al., 2016). 8) Vm24 from *Vaejovis mexicanus smithi* venom reduces delayed-type hypersensitivity reactions in rats (Varga et al., 2012), as well as impairs the synthesis and secretion of T cell cytokines in response to T-cell receptor engagement (Veytia-Bucheli et al., 2018).

Some reports have shown that maurocalcine from the scorpion *Maurus palmatus*, a toxin active on ryanodine receptors, goes into the cells and can also be used as a vector for the penetration of cell-impermeable cargo molecules. Mutated analogues of maurocalcine have been produced as leads to develop better cell-penetrating peptides (CPPs) (Esteve et al., 2005; Ram et al., 2009). CPPs are short (9-35 residues) cationic or amphipathic molecules with the capability of being rapidly internalized across cell membranes. In this way, they can mediate the translocation of a conjugated drug across plasma membranes, being considered an effective and non-toxic mechanism for drug delivery (Ramsey and Flynn, 2015). The first Ca^2+^ channel toxin from *T. serrulatus* venom, designated as CPP-Ts, exhibited selective internalization properties and specific nuclear delivery, being a potential intranuclear delivery tool to target cancerous cells (de Oliveira-Mendes et al., 2018).

### Sea Anemones

Sea anemones, the polyp form of marine coelenterates of the phylum Cnidaria (Watters, 2005), are poorly studied, but represent a rich source of new compounds. ShK-186, originally isolated from *Stichodactyla helianthus* sea anemone venom, inspired the design of dalazatide, a synthetic peptide composed of 37 amino acids, acting as a K_v_1.3 inhibitor (Beeton et al., 2006). In preclinical tests, dalazatide have significantly reduced the clinical score of rat model of multiple sclerosis (Tarcha et al., 2012). Dalazatide completed phase I trials in 2015 to examine the safety of systemic multiple ascending dose administration in 32 healthy volunteers (NCT02446340) and in 24 patients with plaque psoriasis (NCT02435342). No phase II study has been started since then. However, public databases (e.g., the FDA, Drugs.com, etc.) do not mention what happened to this drug lead.

### Snakes

Recently, collinein-1, a SVSP from *Crotalus durissus collilineatus* venom (Boldrini-França et al., 2015) was recombinantly expressed in *Pichia pastoris* system (Boldrini-Franca et al., 2019) and demonstrated to block, independently from its catalytic activity, the hEAG1 ion channel, which is overexpressed in several cell cancer lines. Collinein-1 reduced the viability of human breast cancer cell line MCF7, which displays high expression of hEAG1, but does not affect the HepG2 and MCF10A cell lines, which present low expression of this ion channel, demonstrating that the reduction of cell viability might be connected with hEAG1 inhibition by this protein (Boldrini-França et al., 2020).

Isolated from the Malayan pit viper (*Calloselasma rhodostoma*), ancrod is a thrombin-like enzyme able to release fibrinopeptide A from fibrinogen Aα chain, causing hypofibrinogenemia in humans (Reid, 1971). Structurally, it is composed of 234 amino acids and presents six disulfide bonds (Burkhart et al., 1992). Because of its enzyme activity on fibrinogen (Chan et al., 2016), this toxin was used in stroke treatment (Pizzo et al., 1972), marketed for several decades by Knoll Pharma in Germany and Austria, until it was withdrawn in the 1980s (Chan et al., 2016). In 2002 the rights of this drug were licensed. Two parallel trials (NCT00141001 and NCT00300196) were in phase III of clinical trials by Neurobiological Technologies (NTI), but both studies were terminated due to low efficacy, suboptimal and inconsistent results which led to the dissolution of NTI in 2009 (King, 2011; Liu et al., 2011). A randomized study involving this molecule completed phase II trial in 31 patients with sudden hearing loss to check its effectiveness, safety, and tolerance for this kind of pathology in 2018 (NCT01621256).

The association of 15 residues of the C-terminal portion of *Dendroaspis* natriuretic peptide, isolated from *D. angusticeps* venom, with 22 residues of a human C-type natriuretic peptide, formed the chimeric natriuretic peptide, cenderitide (CD-NP) (Lisy et al., 2008; Lee et al., 2009). It can be applied in congestive heart failure, and its mechanism of action is associated with the connection to natriuretic peptide receptors, leading to hypotension (Wei et al., 1993; Lee et al., 2009). Studies with this peptide completed phase I in 2007, in a non-randomized way, to check its efficacy, safety, and pharmacodynamics in 22 healthy participants (NCT00482937) (Lee et al., 2009; Ichiki et al., 2019). A phase II study in 14 patients with stable chronic heart failure (NCT02359227) and a phase I/II randomized study in 8 patients with chronic stable heart failure and moderate renal impairment (NCT02603614) were completed in February 2020. Another phase I randomized study to maintain the function of left ventricle in 30 participants with myocardial infarction was completed in 2019 (NCT02071602).

Fibrin sealant or fibrin glue, a bioproduct formed by a thrombin-like serine protease from *C. d. terrificus* venom and fibrinogen-rich cryoprecipitate from humans, could transmit infectious diseases and was suspended by the FDA in 1978 (Spotnitz, 2014; Ferreira et al., 2017). To overcome this drawback, the Center for the Study of Venoms and Venomous Animals (CEVAP) at São Paulo State University (UNESP), in Brazil, started studying the aforementioned fibrin sealant using a fibrinogen-rich cryoprecipitate from *Bubalus bubalis* buffaloes blood (Barros et al., 2009; Ferreira et al., 2017), and this bioproduct completed phase I/II of clinical trials with 10 patients in phase I and 30 patients in phase II (Ferreira et al., 2017). The fibrin glue displays adhesive, sealant and hemostatic effects due to its proteolytic activity on fibrinogen, producing fibrin monomers, which forms a clot in the presence of calcium (Barros et al., 2009; Ferreira et al., 2017). Similarly, preliminary studies have been conducted to evaluate the effect of direct application of Vivostat^®^ (autologous fibrin sealant) in controlling cerebral bleeding (Graziano et al., 2015; Graziano et al., 2016).

A 33 kDa-batroxobin from *B. atrox* and *B. moojeni* venoms (Itoh et al., 1987; Earps and Shoolingin-Jordan, 1998) was expressed in *P. pastoris* and exhibited biochemical activities similar to those of native protein (You et al., 2004). The recombinant batroxobin used with a medical adhesive synergistically accelerated hemostasis in the mouse liver and femoral artery models, reducing bleeding time and blood loss. Hemostasis was more rapidly achieved with increasing concentrations of batroxobin (You et al., 2014). Other dressings using collagen and chitosan with recombinant batroxobin also controlled bleeding and improved the hemostatic properties of collagen and chitosan pads used alone (Seon et al., 2017).

An analgesic preparation containing cobratide and oxycodone for cancer-related pain (CN104645312) and a keluoqu tablet preparation method using tramadol hydrochloride, ibuprofen and cobratide (also known as ketongning and cobrotoxin) (CN105769791) have been patented. The anti-nociceptive effects of cobrotoxin (the *N. n. atra* snake venom short-chain post-synaptic α-neurotoxin cobratide) do not involve muscarinic acetylcholine or opioid receptors and the molecule has high affinity for the α_1_ subunit of the nicotinic acetylcholine receptors (nAChR) (Gazerani and Cairns, 2014). Cobratoxin, a long-chain post-synaptic α-neurotoxin isolated from the Thailand cobra (*N. kaouthia*), produces anti-nociceptive and anti-inflammatory effects through decreased production of inflammatory cytokines, for example, TNF-α, IL-1, and IL-2, *via* its high affinity for the α_7_ subtype of nAChR (Gazerani and Cairns, 2014).

RPI-78M (Receptin^®^) and RPI-MN (Pepteron^®^) are detoxified and chemically modified forms of cobratoxin and cobrotoxin, respectively (King, 2011; Harvey, 2014). RPI-78M has 71 amino acid residues with five disulfide bonds and completed phase I of clinical trials for multiple sclerosis, while RPI-MN presents 62 amino acid residues and four disulfide bonds (King, 2011). Both molecules show analgesic applications and present the nAChRs as molecular target (Chan et al., 2016). Although RPI-MN is parenterally administered *via* subcutaneous injection, RPI-78M can be orally administered, since its absorption through the oral mucosa occurs when it is formulated with benzalkonium chloride (Reid and Raymond, 2010). Chemical modifications that detoxify these molecules can alter their affinity to nAChRs. They may include their oxidation with ozone, formate (also known as methanoate) and hydrogen peroxide, being the latter more adopted (Reid, 2007). RPI-MN has also completed preclinical studies against Human Immunodeficiency Virus (HIV), protecting cells due to its ability to inhibit viral replication (Reid, 2007; King, 2011; Harvey, 2014). However, its mechanism of action has not been elucidated yet (Reid and Raymond, 2010).

Crotamine, a highly cationic and cysteine-rich CPP from *C. d. terrificus* snake venom, displays membrane translocation capabilities, penetrates into the cell and presents cytoplasmatic, vesicular, and nuclear distribution (Kerkis et al., 2004). This toxin is specifically uptaken by actively proliferating cells, being able to permeate several lineages *in vitro* (Nascimento et al., 2007). Additionally, several molecules based on crotamine structure, including fluorescent derivatives (Tansi et al., 2019) and functionalized with gold nanoparticles (Karpel et al., 2018), for instance, are being developed. Crotamine and its analogues have been tested in healthy and tumorous cell lines, and the results indicate they can be used as selective delivery tools of anticancer molecules (Mambelli-Lisboa et al., 2018).

Another component isolated from *C. d. terrificus* venom is crotalphine (Konno et al., 2008), a potent analgesic comprised of 14 amino acid residues. It acts at peripheral opioid receptors (Gutierrez et al., 2008) and selectively targets TRPA1 ion channels (Bressan et al., 2016), being more potent under conditions of acute peripheral sensitization (Zambelli et al., 2014). The potent and long lasting opioid-mediated antinociception of crotalphine has been evaluated in cancer pain (Brigatte et al., 2013).

### Spiders

The toxin π-theraphotoxin-Pc1a or π-TRTX-Pc1a (also known as psalmotoxin 1 or PcTx1), obtained from the *Psalmopoeus cambridgei* (Trinidad chevron tarantula) venom, is considered a novel therapeutic molecule for treating pain (Monge-Fuentes et al., 2018). Classified as a specific inhibitor of ASIC1a, the most abundant acid-sensing ion channel, the toxin π-TRTX-Pc1 demonstrated an effective analgesic effect comparable to morphine in rat models of acute pain (Mazzuca et al., 2007). Recently, PcTx1 was also reported as a valuable tool for understanding the functional role of ASIC2a heteromeric channels (ASIC1a/2a) (Liu et al., 2018) and had no effect on acid-induced transient or chronic hyperalgesia in a mouse model of fibromyalgia (Chang et al., 2019).

Hi1a, a PcTx1-related toxin isolated from the Australian funnel-web spider *Hadronyche infensa*, partially inhibits ASIC1a and does not affect ASIC1b (Maatuf et al., 2019). This toxin strongly attenuates brain damage after stroke and could be used to protect the brain from ischemic injury (Chassagnon et al., 2017), being considered as a lead for development of neuroprotective agents (Ren et al., 2018).

Purotoxin-1 (PT1), obtained from the central Asian spider *Geolycosa* sp. venom, has also been studied to pain treatment (Monge-Fuentes et al., 2018). Characterized as a specific antagonist of P2X3 purinergic receptor, which is the most-studied subtype of P2X receptor related to pain, PT1 was able to inhibit nociceptive effect in different rat pain models (Grishin et al., 2010).

*Phoneutria nigriventer* (the armed spider) presents different toxins with potential pharmaceutical application and under preclinical tests (Peigneur et al., 2018). The toxin Phα1β, classified as P/Q- and N-type voltage-gated calcium channel blocker, and its recombinant form (produced in *E. coli*) demonstrated analgesic effects in rodent models of pain (Souza et al., 2008). Recently, the same toxin (Phα1β) together with PhTx3-3 (also a voltage-gated calcium channel blocker) demonstrated significant inhibitory effects on the proliferation and viability of different glioma cell lines (M059J, U-138MG and U-251MG) at low concentrations (0.3-100 pM). In the same study, Phα1β and its recombinant form named CTK 01512-2 caused significant reductions of tumor areas *in vivo* using mouse glioblastoma model (Nicoletti et al., 2017). Moreover, Phα1β and its recombinant version were able to reduce the inflammatory phase of the formalin-induced nociceptive behavior in rats, to decrease neuropathic pain caused by chronic constriction injury of sciatic nerve in rats, and to reduce the hyperalgesia caused by melanoma cancer model in mice (Rigo et al., 2017).

*P. nigriventer* venom became attractive because of its induced-priapism effect. The δ-ctenitoxin-Pn2a toxin, also known as δ-CNTX-Pn2a or Tx2-6, modulates voltage-gated sodium (Na_v_) channels and demonstrated an erectile effect in rats (12 µg/kg, subcutaneous or intravenous injection) (Nunes et al., 2008). Interestingly, a minimum dose of 0.006 µg/kg directly injected into the corpus cavernosum can cause erection in mice (Andrade et al., 2008). A synthetic 19-amino acid peptide, PnPP-19, designed from active core of PnTx2-6 tertiary structure, potentiated erection *in vivo* and *ex vivo via* the nitric oxide/cyclic guanosine monophosphate pathway. PnPP-19 is a promising candidate for erectile dysfunction treatment in patients that do not respond to the usual therapies (Silva et al., 2015). Biozeus Biopharmaceutical S.A performed pilot tests with the topical peptide (renamed BZ371) on healthy human beings and has been performing a pilot test with voluntary men with erectile dysfunction associated to hypertension or diabetes. The regulatory toxicological preclinical tests have already started. The next steps involve the final marketing formulation and future clinical trials (Phases 1 and 2) (Biozeus Biopharmaceutical SA, 2018; Johnson & Johnson, 2019){Johnson & Johnson, 2019, Champions of Science® Storytelling Challenge: Latin America and Caribbean Edition;Biozeus Biopharmaceutical SA, 2018, First clinical trial sponsored by Biozeus concluded!}.

Although antimicrobial activities with spider toxins are well documented (for detail see Spider Toxin Database, http://arachnoserver.org), their therapeutic use are limited due to their susceptibility to proteolysis.

### Tetraodontiformes

Tetrodotoxin (TTX), a guanidinium neurotoxin with high affinity for voltage-gated sodium (Na_v_) channels, had traditionally been known for many years as the main toxin from Tetraodontidae pufferfish (Lago et al., 2015). However, the toxin was present not only in other marine animals such as octopuses, gobies and sea stars, but also in phylogenetically unrelated terrestrial and aquatic organisms, including a dinoflagellate *Alexandrium tamarense*, red calcareous algae, arthropods, echinoderms, molluscs, worms, newts, frogs, and bacteria *Actinomyces*, *Aeromonas*, *Alteromonas*, *Bacillus* and *Pseudomonas* (Lago et al., 2015; Assuncao et al., 2017). The blockage of Na^+^ into the cell inhibits the action potentials’ propagation in the excitable cell membranes, which causes neuromuscular paralysis (Duran-Riveroll and Cembella, 2017). TTX has been used for the development of analgesic and anesthetic drugs (Assuncao et al., 2017; Vetter et al., 2017) and, under the trade name Tectin^®^ (Wex Pharmaceuticals Inc.), proceeded to phase III of the clinical trials for the treatment of pain resulting from chemotherapy treatment in 2018 (NCT01655823).

### Other Animals

The salivary secretion from different animals, such as bats, leeches, lizards, shrews and ticks are considered important sources of biologically active compounds. Other animals, such as caterpillars, have biologically active compounds in their bristles. Many of these compounds are still underexploited, lacking information on their chemical structure, physiological role and therapeutic application. Thus, the study of these compounds increases the chances of discovering new compounds with great pharmaceutical potential. The subsections ***Bats*** to ***Ticks*** will address some potential therapeutic molecules found in the saliva or bristles of these animals.

#### Bats

Desmoteplase, also known as “*Desmodus rotundus* salivary plasminogen activator” (DPSA), is a thrombolytic agent for acute ischemic stroke derived from vampire-bat saliva (Medcalf, 2012; Shi et al., 2016). This fibrin-dependent plasminogen activator is composed of 441 amino acids with high fibrin specificity, long half-life, low bleeding tendency, nonactivation by β-amyloid and lack of neurotoxicity (Medcalf, 2012; Shi et al., 2016; Li et al., 2017). A phase III randomized study in 492 participants with acute ischemic stroke was completed in 2015. Currently, there is no drug based on desmoteplase available for commercialization.

#### Caterpillars

Caterpillars from different South American countries, such as Venezuela, Brazil, French Guyana, Peru, Paraguay, Argentina and Colombia, are responsible for a severe bleeding syndrome in humans who touch their bristles (Arocha-Pinango and Guerrero, 2001). *Lonomia obliqua* is the main species of caterpillar found in Southern Brazil and its venom is comprised of molecules with antiviral, procoagulant, fibrinolytic and wound healing activities (Veiga et al., 2005; Reis et al., 2006; Alvarez-Flores et al., 2011; Carmo et al., 2015; Sato et al., 2016). Their toxic compounds are found in the bristle extract, hemolymph, cryosecretion (a crude venomous fluid ejected by the whole secretory tegument of caterpillars, stored at -20°C for 24 h) and tegument extract (Pinto et al., 2006; Veiga et al., 2009). Some compounds with potential therapeutic applications were identified in *Lonomia* sp, e.g. prothrombin or factor X activators, such as Lopap (*L. obliqua* prothrombim activator protease) and Losac (*L. obliqua* Stuart factor activator protease), PLA_2_-like, proteases, hyaluronidases, α-fibrinogenases (e.g. Lonofibrase), protease inhibitors, serpins, lipocalins, and lectins (Veiga et al., 2009). Currently, there are three clinical studies on caterpillars recorded at the Clinical Trials website (US National Library of Medicine, 2020). However, these studies are related to their use as a source of protein in the diet and none of them involves the genus *Lonomia*.

#### Leeches

Two phase II randomized studies involving leech therapy in 118 (NCT00435773) and 60 participants (NCT02612974) with knee osteoarthritis were completed in 2007 and 2015, respectively. Additional information on FDA-approved hirudin analogues from *H. medicinalis* leech saliva and hirudotherapy was reported in section *Achievements With Animal Toxin-Based Molecules*.

#### Lizards

Exenatide is the synthetic version of the native peptide exendin-4 isolated from the saliva of Gila monster lizard (*H. suspectum*) (Eng et al., 1992; Furman, 2012). According to the Clinical Trials website, there are more than 300 clinical studies about exenatide. So far, there are 207 completed studies, 12 terminated, 25 whose status has not changed for 2 years, 47 recruiting volunteers, 11 that are not yet recruiting, and three enrolled by invitation. Among the clinical studies, exenatide has been used in patients with Parkinson’s disease, showing beneficial effects on nerve cells by slowing down or stopping the degenerative process of this disease (NCT03456687). For an extensive review regarding clinical trials involving this drug, please see (Odegard and Desantis, 2009; Bhavsar et al., 2013). Additional information on exenatide was described in section *Achievements With Animal Toxin-Based Molecules*.

#### Shrews

SOR-C13 is a synthetic selective peptide inhibitor of Transient Receptor Potential Vanilloid 6 (TRPV6) calcium oncochannel (Pennington et al., 2017; Soricimed, 2018). It is comprised of 13 amino acids derived from the C-terminal region of the paralytic peptide soricidin (UniProtKB—P0C2P6), from the submaxillary and sublingual salivary glands of the Northern Short-tailed shrew (*Blarina brevicauda*) (Bowen et al., 2013; Fu et al., 2017). It inhibits the activation of nuclear factor of activated T-cell (NFAT) transcription complex, and induces apoptosis in TRPV6-overexpressing cells (NIH National Cancer Institute, 2018). A phase I of study (NCT01578564) in 23 advanced cancer patients with TRPV6 channel overexpression was completed in 2016 and a phase I study (NCT03784677) started recruiting patients with advanced refractory solid tumors in 2019.

#### Ticks

There are several studies addressing the importance of tick saliva components. The use of evasins in the treatment of heart diseases, such as myocarditis (Singh et al., 2017) and the use of ixolaris, an anticoagulant protein from *Ixodes scapularis* tick saliva, to reduce HIV-driven coagulopathy, for instance (Schechter et al., 2017).

However, although tick saliva contains many components with therapeutic and biotechnological potentials, there are neither clinical studies involving the use of substances isolated from tick saliva nor drugs available for therapeutic purposes. The 101 clinical studies currently registered on the Clinical Trial website in respect to ticks are related to the development of vaccines against ticks or the use of different antibiotics in Lyme disease.

## Filling the Gap Between the Drug Discovery and Its Commercialization—Future Trends

Animal poisons and venoms are comprised of a cocktail of bioactive components with a gamut of different activities. Company pipelines worldwide are expanding the number of peptide-based products currently in development mainly because of the diversity of their application and activity. However, industrial production of toxin-related drugs from natural sources is quite challenging, laborious and presents restricted yield (Boldrini-França et al., 2017). To overcome these limitations, the main options are the chemical synthesis of peptides and the production of biopharmaceuticals *via* heterologous expression using biotechnological tools.

Recent data reinforces the advances in transcriptomics, proteomics and heterologous expression techniques, which allowed the characterization and potential production of low abundant active venom components, presenting low or high molecular mass (Boldrini-França et al., 2017). Additionally, pharmacomics has been gaining ground by integrating “omic” approaches to study dynamic molecular states and monitors disease states and drug responses, improving the development of novel drugs (Wilson and Daly, 2018).

The industry has focused on heterologous expression systems as an interesting alternative for manufacturing biopharmaceuticals of high molecular mass (Merlin et al., 2014). Recombinant protein production processes require extensive design and regulatory control before therapeutic products become commercially available. Regarding heterologous expression, the accurate cysteine bond formation and the proper incorporation of post-translational modifications remain a challenge, and new technologies to assess and mitigate immunogenicity risk of engineered proteins are becoming more common. Therefore, a special attempt should be made to ensure that the recombinant protein presents comparable three-dimensional folding and consistent pharmacological properties when compared to its corresponding native form.

Native chemoselective reaction has been employed in the production of animal toxins with potential therapeutic application, such as mambalgin-2, a 57-amino acid analgesic peptide from three-finger toxins family, from *Dendroaspis polylepis polylepis* venom (Diochot et al., 2012; Harvey, 2014). This approach allows the synthesis of large proteins, since it is based in the production of different unprotected linear peptide fragments, which are condensed in solution *via* chemoselective reactions to originate the entire polypeptide (Kent et al., 2012). Studies to improve the protecting groups, resins, linkers, and activation and coupling reagents may enable the manufacture of larger peptides and even small proteins for therapeutic applications. However, the development of cheaper reagents and methods for the synthesis and purification of peptides are necessary.

Concerning the limitations of peptides in terms of their biopharmaceutical properties, designed approaches that will find molecules with intrinsically more favorable properties will need to be devised. As mentioned earlier in section *Achievements With Animal Toxin-Based Molecules*, the drug design of captopril made oral administration possible. Additionally, designed cationicity-enhanced analogues of natural antimicrobial peptides have exhibited higher potency and spectra of antimicrobial activity (Luna-Ramirez et al., 2017).

Achievements towards successful oral delivery of proteins and peptides by protecting them against degradation and increasing their absorption remain as an active area of research. Regarding toxin-based formulations, intranasal inoculation of hyaluronidase from *T. serrulatus* venom induced mononuclear increase in the bronchoalveolar space and became a promising tool for the treatment of pulmonary fibrosis (Bitencourt et al., 2011).

Some approaches to improve biopharmaceuticals delivery, such as alternative delivery routes, PEGylation and conjugation to (nano)carriers, represent a relevant step towards targeted delivery of toxin-based drugs. It is sobering to realize how little alternative delivery routes and bioconjugation strategies have been exploited to deliver toxin-based drugs, suggesting that studies on routes of distribution, delivery vehicles, cargo molecules, and targeting strategies are fruitful fields for future research.

Collinein-1, a thrombin-like serine protease from *C. d. collilineatus*, was successfully modified by site-specific PEGylation with maleimide-mPEG of 5 kDa and exhibited higher catalytic efficiency and affinity for the substrate than the native form (da-Silva-Freitas et al., 2015). The PEGylated peptide HsTX1[R14A] from *Heterometrus spinnifer* scorpion venom showed higher plasma circulating half-life in rodents compared to the native peptide, which resulted in sustained efficacy in rodent models of multiple sclerosis and rheumatoid arthritis (Tanner et al., 2017).

In the drug development process, formulation patents using advanced drug release systems extend the market exclusivity of drugs, because of the high technical barrier to be overcome by generic manufacturers after the expiration of patents. Focusing on competitiveness, pharmaceutical companies have established strategic partnerships with leading academic institutions that have deep scientific expertise in novel concepts in the main areas of biology or chemistry. Some reports have shown the antitumor potential, among other applications, of animal venoms or their toxins conjugated with a wide variety of nanomaterials, such as silica, gold, chitosan, poly(D,L-Lactide)-based, and supermagnetic iron oxide nanoparticles (Badr et al., 2014; Utkin, 2017).

Studies on cell penetrating peptides (CPPs) have open unprecedented possibilities for vector applications in several fields, such as basic research, therapeutics, technology, and medical imaging. CPP-Ts, the first Ca^2+^ channel toxin from *T. serrulatus* venom, showed to be a potential intranuclear delivery tool to target cancerous cells (de Oliveira-Mendes et al., 2018). WaTx, a cell-penetrating toxin from the Australian black rock scorpion *U. manicatus*, reduced the permeability of the TRPA1 ion channel to Ca^2+^ ions and can be used as a tool to study the mechanisms involved in chronic pain (King et al., 2019).

Another field in ever-growing demand is the cosmeceutical industry, especially in Asia, where Korea is at the forefront of cosmeceutical development. Efficacy and safety studies on these products in humans are on high demand (Juhász et al., 2018).

## Conclusion

**Figure 2:**
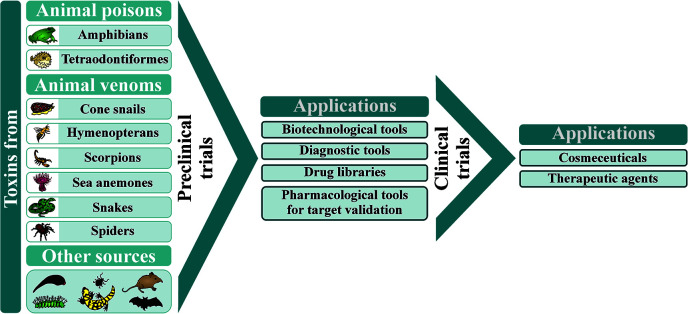
Animal poisons and venoms as sources of candidate molecules for wide-ranging applications, after extensive characterization during preclinical and clinical trials.

Animal poisons and venoms are rich sources of molecules with a wide range of applications. However, to make the use of these molecules feasible, extensive preclinical trials are necessary, with some applications also requiring clinical trials (**Figure 2**).

Although the research in the field of toxinology tends to be quite challenging and time-consuming, the high selectivity of animal toxins for their targets turns them into promising leads for the development of effective therapeutic drugs. Studies on new engineered molecules with reduced side effects can be reached by untangling the interaction of venom peptides with their target. Therefore, we are still at a beginning phase in comprehending the complexity of animal venoms and poisons. While very few species have been extensively studied, we still have thousands of unexploited organisms, especially marine ones. Novel methods to produce and deliver biopharmaceuticals are expected to be developed in the near future. With that in mind, we can get a glimpse of how much work on toxinology and drug discovery is yet to come in the next years.

## Author Contributions

KB and EA contributed to the conception and design of the study. KB and EP-J wrote the first draft of the manuscript. KB, CC, EF-B, EP-J, FCe, FGA, FPA, FCo, GW, IC, IF, IO, JB-F, MP, MB, and EA wrote sections of the manuscript. FCe created the figures. KB, GW, IC, and IO created the tables. All authors contributed to manuscript revision, read, and approved the submitted version.

## Funding

This study was supported by the São Paulo Research Foundation (FAPESP, grants 2017/04724-4 and 2019/10173-6, and scholarships to CC 2013/26200-6, EP-J 2016/04761-4; FCe 2017/14035-1 and 2018/14158-9; GW 2017/00586-6; ISO 2017/03580-9 and 2018/21233-7; KB 2013/26619-7), the National Council for Scientific and Technological Development (CNPq, grants 306479/2017-6 and 307155/2017-0 and scholarships to FCo 155276/2018-2 and FGA 150037/2018-0) and the Coordination for the Improvement of Higher Education Personnel (Coordenação de Aperfeiçoamento de Pessoal de Nível Superior—Brasil, CAPES, Finance Code 001, scholarships to EP-J 88881.186830/2018-01, GW, IF, IO, and JB-F).

## Conflict of Interest

The authors declare that the research was conducted in the absence of any commercial or financial relationships that could be construed as a potential conflict of interest.
